# Redox-sensitive doxorubicin liposome: a formulation approach for targeted tumor therapy

**DOI:** 10.1038/s41598-022-15239-x

**Published:** 2022-07-04

**Authors:** Elaheh Mirhadi, Mohammad Mashreghi, Anis Askarizadeh, Amin Mehrabian, Seyedeh Hoda Alavizadeh, Leila Arabi, Ali Badiee, Mahmoud Reza Jaafari

**Affiliations:** 1 Nanotechnology Research Center, Pharmaceutical Technology Institute, Mashhad University of Medical Sciences, Mashhad, Iran; 2 Department of Pharmaceutical Nanotechnology, School of Pharmacy, Mashhad University of Medical Sciences, Mashhad, Iran

**Keywords:** Cancer, Cancer, Chemical biology, Drug discovery, Chemistry, Drug development

## Abstract

In this study redox-sensitive (RS) liposomes manufactured using 10,10′-diselanediylbis decanoic acid (DDA), an organoselenium RS compound, to enhance the therapeutic performance of doxorubicin (Dox). The DDA structure was confirmed by 1H NMR and LC–MS/MS. Various liposomal formulations (33 formulations) were prepared using DOPE, Egg PC, and DOPC with Tm ˂ 0 and DDA. Some formulations had mPEG_2000_-DSPE and cholesterol. After extrusion, the external phase was exchanged with sodium bicarbonate to create a pH gradient. Then, Dox was remotely loaded into liposomes. The optimum formulations indicated a burst release of 30% in the presence of 0.1% hydrogen peroxide at pH 6.5, thanks to the redox-sensitive role of DDA moieties; conversely, Caelyx (PEGylated liposomal Dox) showed negligible release at this condition. RS liposomes consisting of DOPE/Egg PC/DDA at 37.5 /60/2.5% molar ratio, efficiently inhibited C26 tumors among other formulations. The release of Dox from RS liposomes in the TME through the DDA link fracture triggered by ROS or glutathione is seemingly the prerequisite for the formulations to exert their therapeutic action. These findings suggest the potential application of such intelligent formulations in the treatment of various malignancies where the TME redox feature could be exploited to achieve an improved therapeutic response.

## Introduction

Doxorubicin (Dox) is a common first-line therapy mainly applied alone or in combination with other chemotherapeutics to treat various cancers including ovarian, lung, breast, and bladder^1^. Despite the vast usages, adverse effects associated with Dox including cardiotoxicity, have led to the development of PEGylated liposomal doxorubicin (PLD, Doxil/Caelyx), which incorporate Dox within their aqueous core^2^. Compared to conventional Dox, treatment with PLD results in reduced cardiotoxicity, nausea, vomiting, and myelosuppression^3^. PLDs have shown outstanding priority in delivery of anti-tumor drugs such as Dox compared to other DDSs. Some other DDSs have been investigated including Dox conjugated to a biodegradable dendrimer and evaluated in mice bearing C-26 colon carcinoma. However, these systems have not been successful clinically^4^. PLD improves the accumulation of carriers within the tumor tissue through enhanced permeation and retention (EPR) effect^5^. The fewer side effect of PLD is due to the decreased exposure of normal tissues to Dox. Despite this, the anti-tumor efficacy of PLD is still limited due to low stability, insufficient availability, or uncontrolled and inadequate release of the encapsulated drug at the tumor site^6,7^. Several factors are contributed to the release properties of liposomes including liposome stability within the circulation, liposomal membrane composition, mechanism of drug loading (passive or remote loading) and the tumor microenvironment (TME) pathological features including low pH value, higher levels of glutathione or reactive oxygen species (ROS) and overexpression of various specific enzymes^6,8,9^. Liposomal membrane lipid composition is an important criterion to control the desired drug release pattern^10,11^. The presence of high transition temperature (Tm) hydrogenated soy phosphatidylcholine (53 °C) and a considerable amount of cholesterol in the Caelyx membrane eliminates the solid ordered (SO) to liquid disordered (LD) phase transition at body temperature, mimicking the condition where cholesterol is absent in the lipid bilayer. This membrane demonstrates a prolonged, nearly zero-ordered release rate at 37 °C in vitro in buffers and plasma, as well as in vivo in mice models^12^. Thanks to the incredible potential of nanomedicine, various stimuli-responsive nano-drug delivery systems (NDDS) represented the capability of spatial and temporal control over therapeutic agent release within the TME. Therefore, to trigger drug release at the cancerous tissue, a DDS can be manipulated to respond to an external stimulus (including heat, ultrasound, light, magnetic field) or an endogenous stimulus (including redox potential, pH, and unique enzymatic activity) in the TME^13–16^. In liposomal nanocarriers, stimuli-responsive release of the loaded compounds is generally based on the destabilization of bilayer^17,18^. It has been demonstrated that redox (reduction–oxidation) responsive liposomes could utilize electron-transfer reactions to trigger the release of drug. Glutathione (GSH) and reactive oxygen species (ROS) including superoxide anion (O_2_^**·**−^), hydroxyl radical (HO^**·**^), hydrogen peroxide (H_2_O_2_), etc. can modify the charge and hydrophilic nature of the linkers containing reducing agents or remove the cross-links in functionalized liposomes^17,19^.

TME comprises different types of cells (endothelial cells, fibroblast, cancer-associated fibroblasts, immune cells, and invasive tumor cells), blood vessels, inflammatory factors, and various types of ROS. Myeloperoxidase, superoxide dismutase, NADPH oxidase, and mitochondrial electron transport system are the main sources of ROS generation^20–23^. In a cancer cell, the level of H_2_O_2_ increases by 0.5 nmol/10^4^ cells/h to reach approximately 100 mM, which is 100 times higher compared to that of normal cells^18,24^.

Selenium (Se) is an essential trace element in the human body possesses particular biological functions in the oxidation–reduction system^25^. Selenium-containing compounds have indicated anti-tumor effects in mice bearing colon carcinoma (HCT-8 and HT-29) and human squamous cell carcinoma of the head and neck (FaDu and A253) xenograft model^26^. To enhance the intracellular drug release, NDDSs containing the Se–Se bond (diselenide) have been developed^27,28^. Similar to disulfide (S–S), the diselenide bond is cleaved upon exposure to the redox conditions of the TME. However, the bond energy to cleave the Se–Se bond is lower (172 kJ/mole) compared to that of the S–S bond (240 kJ/mole), leading to easier breakage of diselenide bonds^29^. The presence of oxidants could cleave and oxidize the Se–Se bonds to seleninic acid, while a reducing environment, reduces it to selenol^27^. Using this approach, Zhang et al*.* designed redox responsive micelles based on poly (ethylene glycol)-*b*-polyurethane-*b* poly (ethylene glycol) copolymer composed of diselenide which released the loaded drug at a relatively low GSH concentration^27^. Yan et al*.* also encapsulated Dox in hyperbranched polydiselenide micelles, which showed the EPR effect and inhibited the proliferation of various forms of cancer cells^30^. In another study by Wang et al*.* a redox system was fabricated by polymer-conjugated lipids containing diselenide^31^. Compared with other DDSs composed of diselenide, liposomes exhibit better properties, including site-targeting, sustained or controlled release, protection of drugs from degradation and clearance, superior therapeutic effects, and lower toxic side effects.

**Figure 1:**
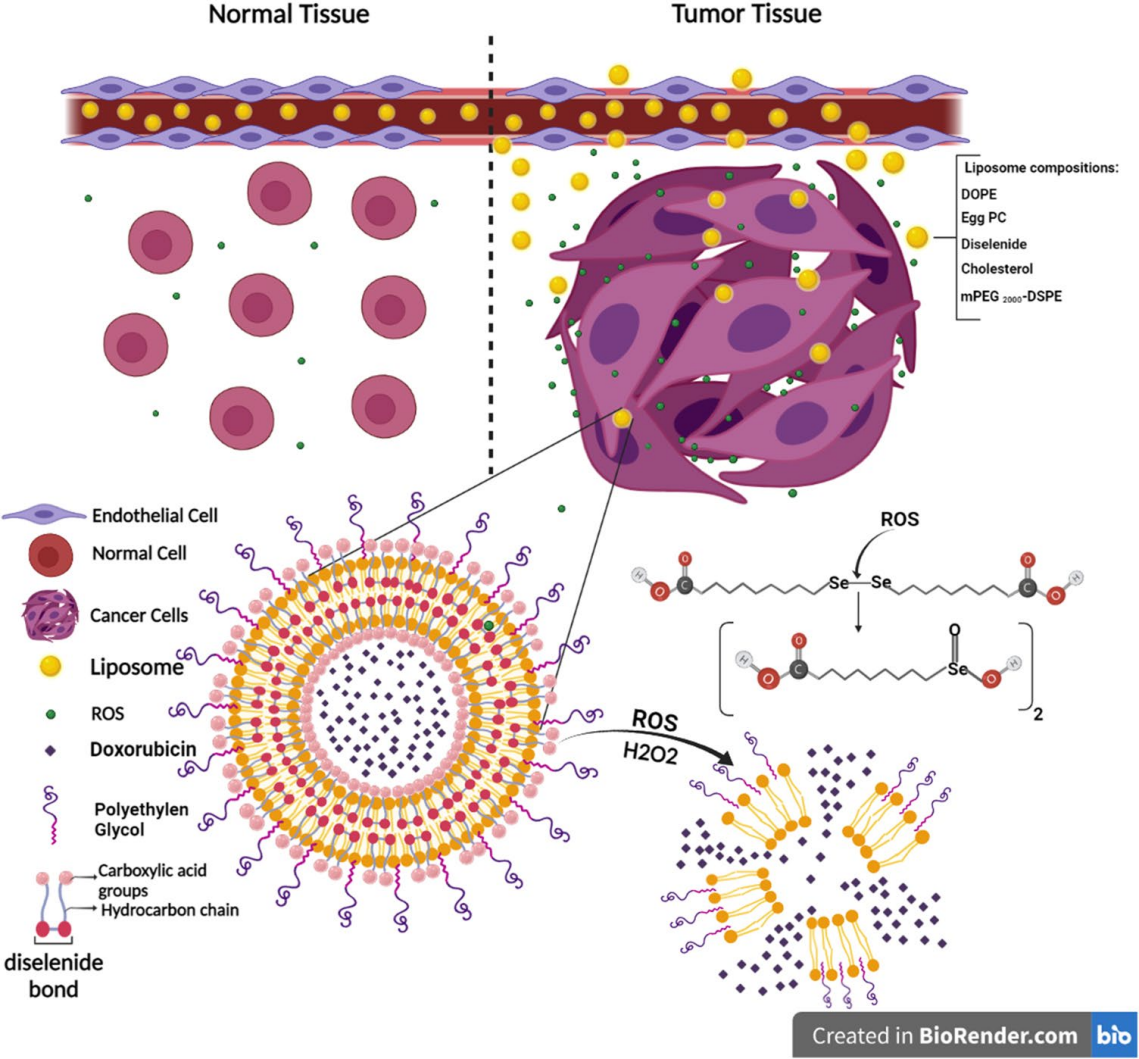
Cleavage of diselenide bond in the presence of ROS in tumor microenvironment and destruction of liposome structure.

Herein, redox sensitive (RS) liposomal formulations were developed, which contained RS amphiphilic organoselenium compound, 10,10′-diselanediylbis decanoic acid (DDA) with diselenide link sensitive to the redox potential of TME (Fig. 1). Further, low Tm phospholipids including Egg PC, DOPE, and DOPC were used as the building blocks of RS liposomes prepared by thin-film hydration, which was then in situ remote loaded with Dox. The formulations with desired size (107.70–223.20 nm) and colloidal stability demonstrated a burst release of 30% in the presence of 0.1% hydrogen peroxide due to the redox-sensitive feature of DDA moieties. RS liposomal formulations improved cellular uptake and showed significant toxicity on murine colon cancer cells (C26 cells). The in vivo study in BALB/c mice bearing C26 tumor indicated a significantly prolonged survival of animals in RS liposomes compared to Caelyx. DOPE/Egg PC/DDA formulation at 37.5/60/2.5% molar ratio efficiently suppressed C26 tumor growth and improved Dox tumor distribution. These features highlight the potential of redox-sensitive engineered liposomes as smart nanomedicine in treating various malignancies.

## Results

### Synthesis of 10,10′-diselanediylbis decanoic acid (DDA)

**Figure 2:**
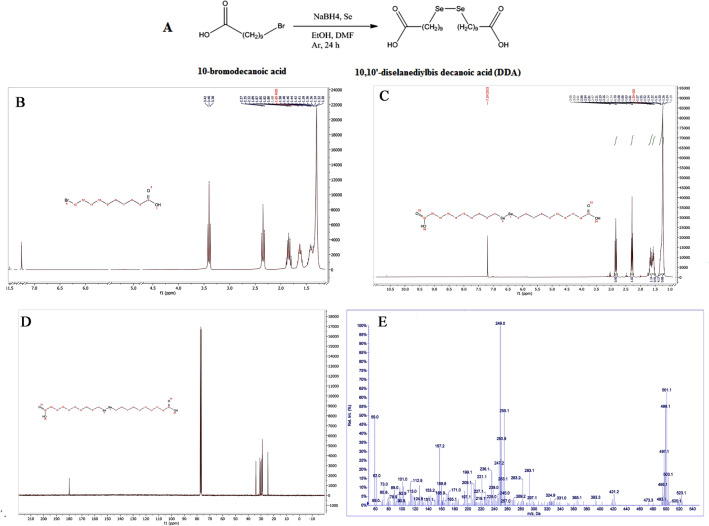
(**A**) The schematic synthesis of 10,10′-diselanediylbis decanoic acid (DDA), (**B**) ^1^H NMR spectra of 10-bromodecanoic acid, (**C**) ^1^H NMR spectra of DDA, (**D**) ^13^C NMR of DDA, (**E**) LC-mass/mass of DDA.

In the first step, synthesis of DDA was successfully conducted through the reaction of 10-bromodecanoic acid and Se powder in the presence of NaBH_4_ as a reducing agent under an inert atmosphere (Fig. 2A). The product was obtained in light yellow color with a melting point of 82 °C, and molecular weight of 500 g/mole. Figure 2C shows ^1^H-NMR spectra of the formation of DDA. The peak of (**–**CH_2_-Br) at 3.4 ppm in 10-bromodecanoic acid has changed to 2.84 ppm in diselenide related to (–CH_2_-Se-) (Fig. 2B,C). C-NMR of the DDA also confirmed the synthesis, via the presence of the peak at 180 ppm related to the carbonyl group (C=O) and the peak of C next to the carbonyl group at 34 ppm (Fig. 2D). In the LC–MS/MS spectrum, Fig. 2E, the peak at 500 *m/z* Da is in accordance with DDA molecular weight (500 g/mol).

### Physicochemical characterization of RS liposomes

To develop the best formulations containing DDA, we started with two-component formulations in which the type and the ratios of the two phospholipids were considered as variables. To enhance the release of Dox from the formulations, phospholipids with lower transition temperature (Tm ˂ 0 °C) were selected, including DOPE, Egg PC, and DOPC. In this way, the M8 formulation showed optimal features in terms of size, PDI, and zeta potential. The next step was the addition of DDA to the formulations. For this, DDA at different percentages (2.5, 5, 7.5, 10, 15, 20) was added to the formulations (M9-M17). The optimum DDA concentration was 2.5% based on the sizes, PDI, and zeta potentials of the formulations following Dox loading.

Then, mPEG_2000_–DSPE was added to the formulations at 1, 2.5, and 5% (M18–M29). The formulations with 5% of mPEG_2000_–DSPE were selected for the next steps. The addition of Chol as the following variable, was applied to enhance the stability and rigidity of the formulations (M30–M33) (Supplementary Table S1).

**Figure 3:**
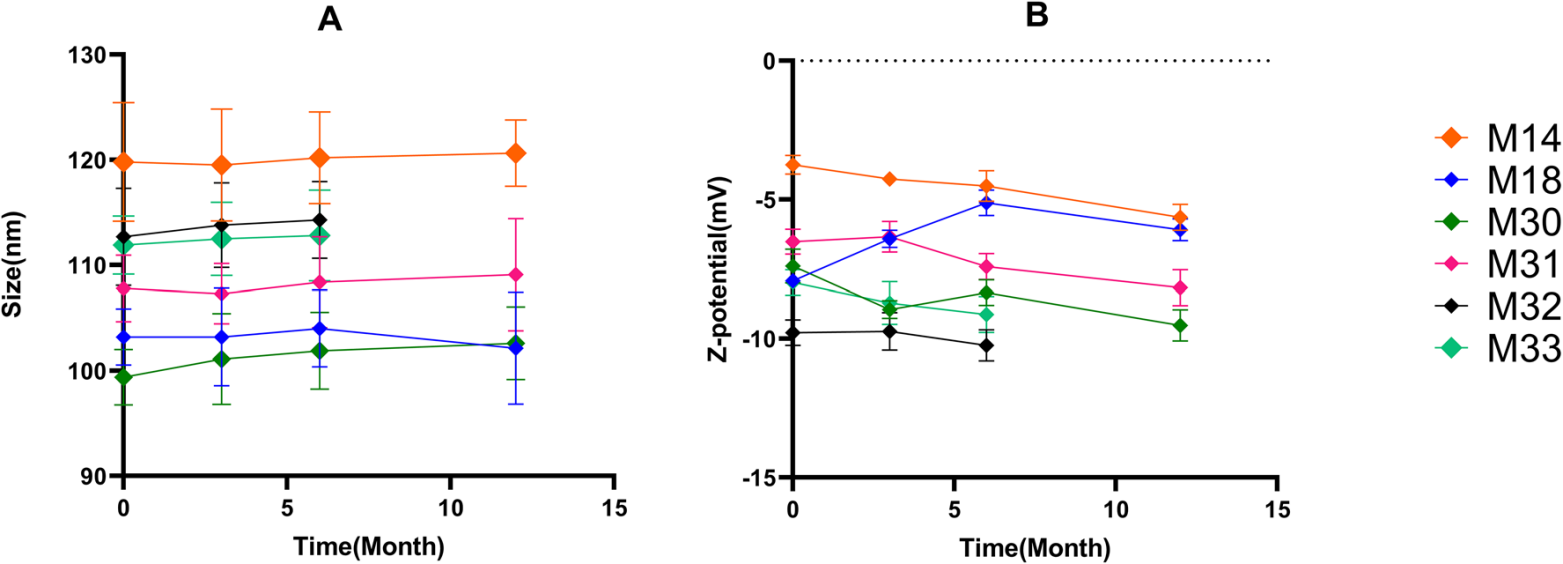
(**A**) The hydrodynamic size of blank RS liposomes measured by DLS after 3,6 and 12 months; (**B**) Z-potential of blank RS liposomes by DLS after 3,6 and 12 months. The data were expressed as mean ± SD (n = 3), P value < 0.0001.

**Figure 4:**
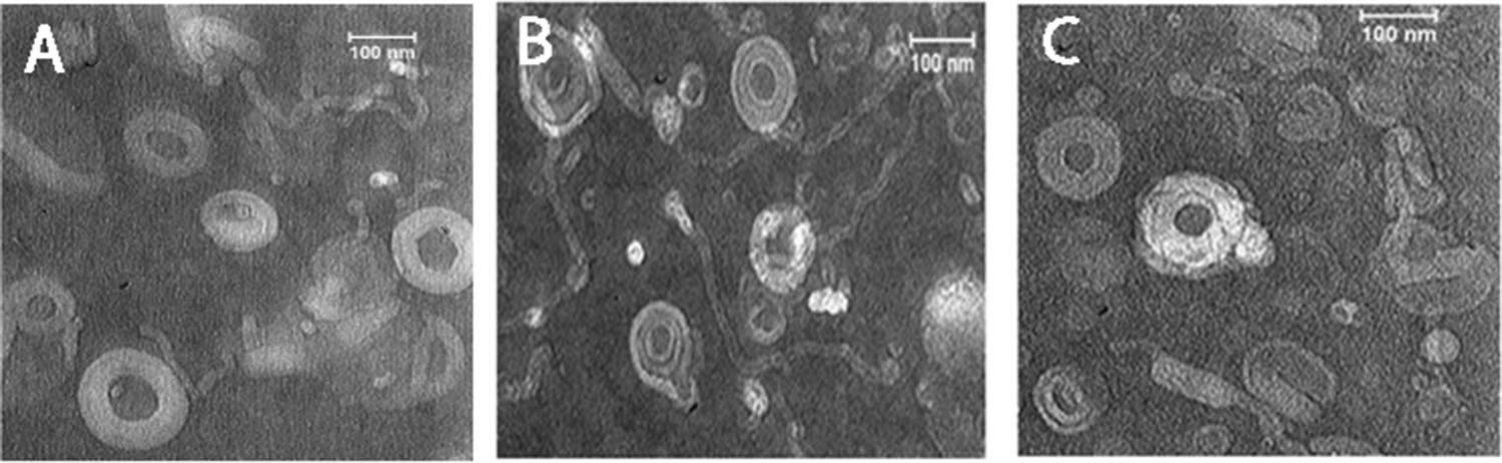
TEM images of (**A**) M14, (**B**) M18, (**C**) M32. Samples are diluted in a ratio of 1:100.

The final formulations were M14 which contained DDA with no mPEG_2000_–DSPE and Chol (DOPE/Egg PC/DDA: 37.5/60/2.5), M18, a formulation without Chol (DOPE/Egg PC/DDA/ mPEG_2000_–DSPE: 37.5/55/2.5/5), and M30–M33 containing different amounts of Chol and DOPE. All properties of the formulations are mentioned in Supplementary Table S1 in the supplementary file after Dox loading. Table 1 shows the characterization of the final formulations before drug loading. Table 2 shows the characterization of the final formulations after in situ loading of Dox into RS liposomes. As shown in Table 1, the size range of RS liposomes was changed from 99.38 to 161.70 before drug loading to 107.70–223.20 nm after drug loading. The zeta potentials of all RS liposomes were ranged from − 3.75 to − 9.78 mV that changed to − 10.30 to − 19.50 mV following Dox loading. M14 formulation showed the largest size and zeta potential. To evaluate the stability of blank-liposomes, the characterization of liposomes, including size, PDI, and zeta potential, were assessed after 3, 6, and 12 months. The results exhibited that all liposomes were stable and free of precipitation or aggregation with only negligible changes in the zeta potential and size distribution after storage at 4 °C. Figure 3, indicates their relatively high stability. In Fig. 4, the transition electron micrographs of M14, M18, and M32 are shown.

**Table 1:** Final RS formulations before in situ loading of Dox.

Name	Formulation	PDI^a^ ± SD^b^	Z-average ± SD (nm)	Intensity ± SD	Volume ± SD	Number ± SD	Zeta potential ± SD (mv)
M14	DOPE/Egg PC/Se-Se 37.5/60/2.5	0.148 ± 0.01	161.70 ± 3.46	157.20 ± 4.51	146.30 ± 5.46	114.80 ± 4.83	− 3.75 ± 0.06
M18	DOPE/Egg PC/Se-Se/mPEG 37.5/55/2.5/5	0.07 ± 0.01	103.20 ± 4.21	113.50 ± 5.32	96.32 ± 6.24	77.42 ± 5.43	− 7.92 ± 0.08
M30	DOPE/Egg PC/Se-Se/mPEG/Chol 27.5/55/2.5/5/10	0.098 ± 0.021	99.38 ± 6.41	110.20 ± 5.75	90.41 ± 8.32	70.58 ± 3.68	− 7.39 ± 1.21
M31	DOPE/Egg PC/Se-Se/mPEG/Chol 17.5/55/2.5/5/20	0.087 ± 0.03	107.80 ± 7.21	117.80 ± 8.14	101.30 ± 7.53	80.91 ± 6.94	− 6.52 ± 0.086
M32	DOPE/Egg PC/Se-Se/mPEG/Chol 7.5/55/2.5/5/30	0.063 ± 0.024	112.70 ± 4.65	121.50 ± 7.12	107.60 ± 6.41	88.48 ± 5.61	− 9.78 ± 0.073
M33	DOPE/Egg PC/Se-Se/mPEG/Chol 0/55/2.5/5/37.5	0.120 ± 0.019	111.90 ± 5.12	125.30 ± 4.43	104.90 ± 3.96	79.58 ± 5.76	− 7.98 ± 0.096

a, Poly dispersity index.

b, Standard deviation.

**Table 2:** Final RS liposomal formulations after in situ loading of Dox.

Name	Formulations contents (%)	PDI^a^ ± SD^b^	Z-average ± SD (nm)	Intensity ± SD	Volume ± SD	Number ± SD	Zeta Potential ± SD (mv)
M14	DOPE/Egg PC/Se-Se 37.5/60/2.5	0.231 ± 0.04	223.20 ± 6.57	262.50 ± 7.28	286.50 ± 6.14	141.43 ± 5.43	− 19.50 ± 0.05
M18	DOPE/Egg PC/Se-Se/mPEG 37.5/55/2.5/5	0.088 ± 0.002	112.20 ± 5.91	123.50 ± 6.43	102.32 ± 4.87	87.42 ± 5.16	− 7.92 ± 0.031
M30	DOPE/Egg PC/Se-Se/mPEG/Chol 27.5/55/2.5/5/10	0.118 ± 0.01	107.70 ± 6.24	120.70 ± 5.64	100.30 ± 7.14	76.60 ± 5.96	− 11.30 ± 0.04
M31	DOPE/Egg PC/Se-Se/mPEG/Chol 17.5/55/2.5/5/20	0.130 ± 0.009	117.40 ± 7.48	120.50 ± 6.74	108.40 ± 5.98	92.15 ± 6.45	− 11.00 ± 0.042
M32	DOPE/Egg PC/Se-Se/mPEG/Chol 7.5/55/2.5/5/30	0.102 ± 0.008	123.70 ± 8.21	137.90 ± 7.56	119.50 ± 6.84	98.64 ± 7.46	− 10.30 ± 0.051
M33	DOPE/Egg PC/Se-Se/mPEG/Chol 0/55/2.5/5/37.5	0.141 ± 0.007	132.80 ± 6.85	147.30 ± 7.41	118.00 ± 8.12	100.66 ± 5.63	− 13.10 ± 0.034

^a^Poly dispersity index.

^b^Standard deviation.

### In vitro release of Dox from RS liposomes

#### In vitro release of Dox in various pH (5.5, 6.5, 7.4)

**Figure 5:**
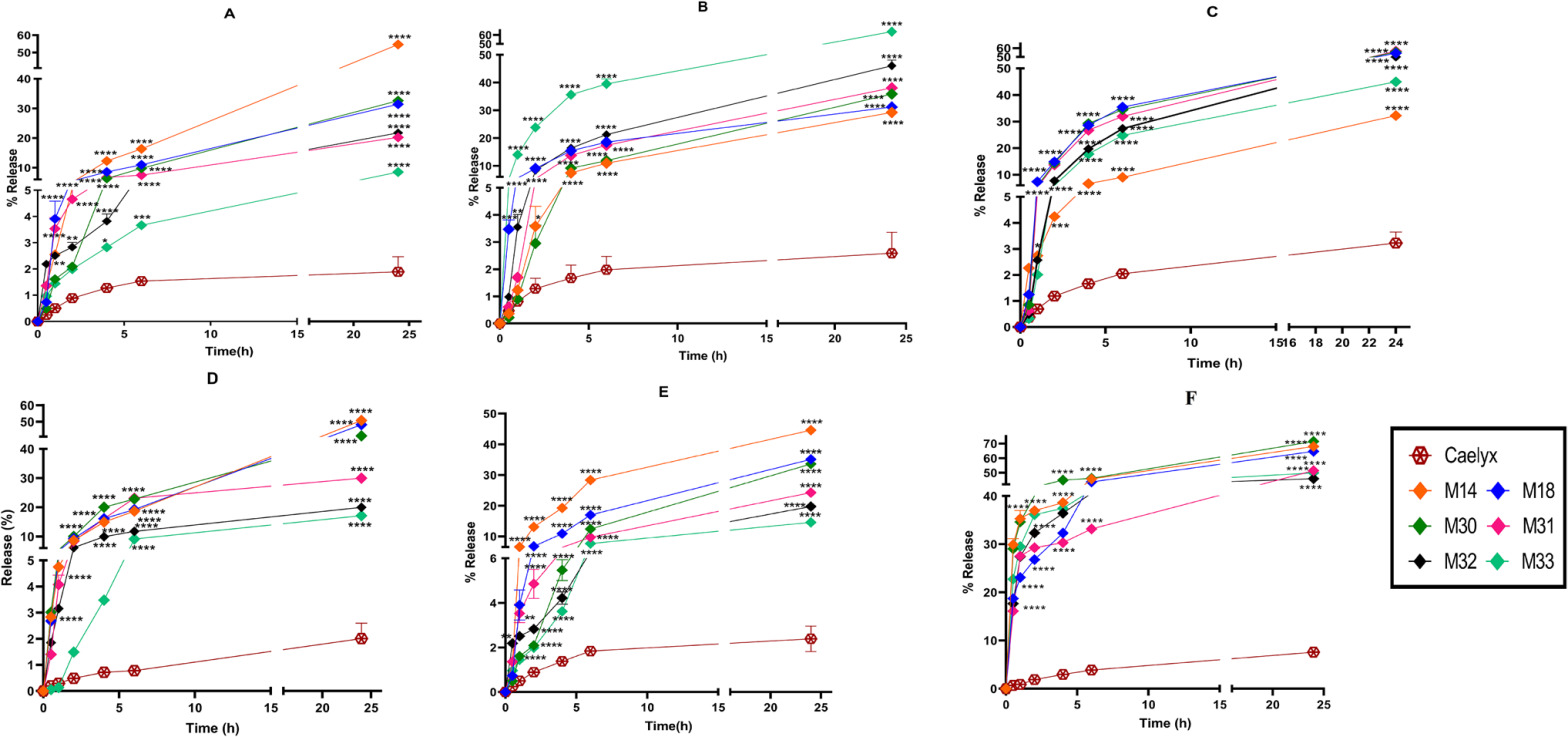
In vitro release of Dox from RS liposomal formulations. (**A**) The release of all formulations containing Dox in Phosphate buffer (pH: 7.4), (**B**) In phosphate buffer (pH: 6.5), (**C**) In succinate (pH: 5.5), (**D**) In plasma and (**E**) The release of RS liposomal formulations containing pyrvinium phosphate in Phosphate buffer (pH: 7.4), (**F**) The release of RS liposomal formulations containing pyrvinium phosphate in phosphate buffer (pH: 6.5) in presence of 0.1% H_2_O_2_. Data are means ± SD (n = 3).

The cumulative release of Dox from RS liposomal formulations was evaluated by dialysis method at 37 °C. As shown in Fig. 5, Caelyx had the lowest release during 24 h. In phosphate buffer, pH: 7.4, M14 showed the highest release of Dox (55%) and M33 with the highest Chol ratio among other formulations, resulted in the lowest Dox release (8.55%) (Fig. 5A). There was no significant difference between RS formulations and Caelyx at 0, 15, 30, 45 min time points. After 1 h, however, Dox release from RS liposomes significantly increased compared to Caelyx, which continued up to 24 h incubation time. As shown in Fig. 5B, C, at lower pH (6.5 and 5.5), the release of Dox from the M14 formulation was only 29 and 32.2%, respectively. Comparing the Dox release in plasma also demonstrated the lowest release with M32 and M33 formulations (less than 20%) (Fig. 5D). The considerably higher release of Dox from RS liposomes compared to Caelyx at pH 5.5 and in plasma after 30 min highlights the favorable release properties of RS formulations (p ˂ 0.0001).

#### In vitro release of formulations in the presence of 0.1% H_2_O_2_

Due to the oxidation potential of Dox in the presence of H_2_O_2_, pyrvinium phosphate was loaded into the formulations to evaluate the release of cargo from RS liposomes. Characterization of RS liposomes containing pyrvinium phosphate is shown in Supplementary Table S2 in the supplementary file. As shown in Fig. 5F, for all formulations, a burst release has been occurred (more than 30%) with a significant difference compared to Caelyx (p ˂ 0.001). While this burst release was not observed at pH 7.6 (Fig. 5E). The formulations with the highest Chol ratio (M31, M32, M33) showed a lower release rate than those with lower Chol content (M14, M18, and M30). Supplementary Figure S1, shows Dox oxidation in the presence of H_2_O_2_ in 24 h (two peaks are appeared from the first half-hour of exposure and is continued during 24 h).

### In vitro cytotoxicity

**Figure 6:**
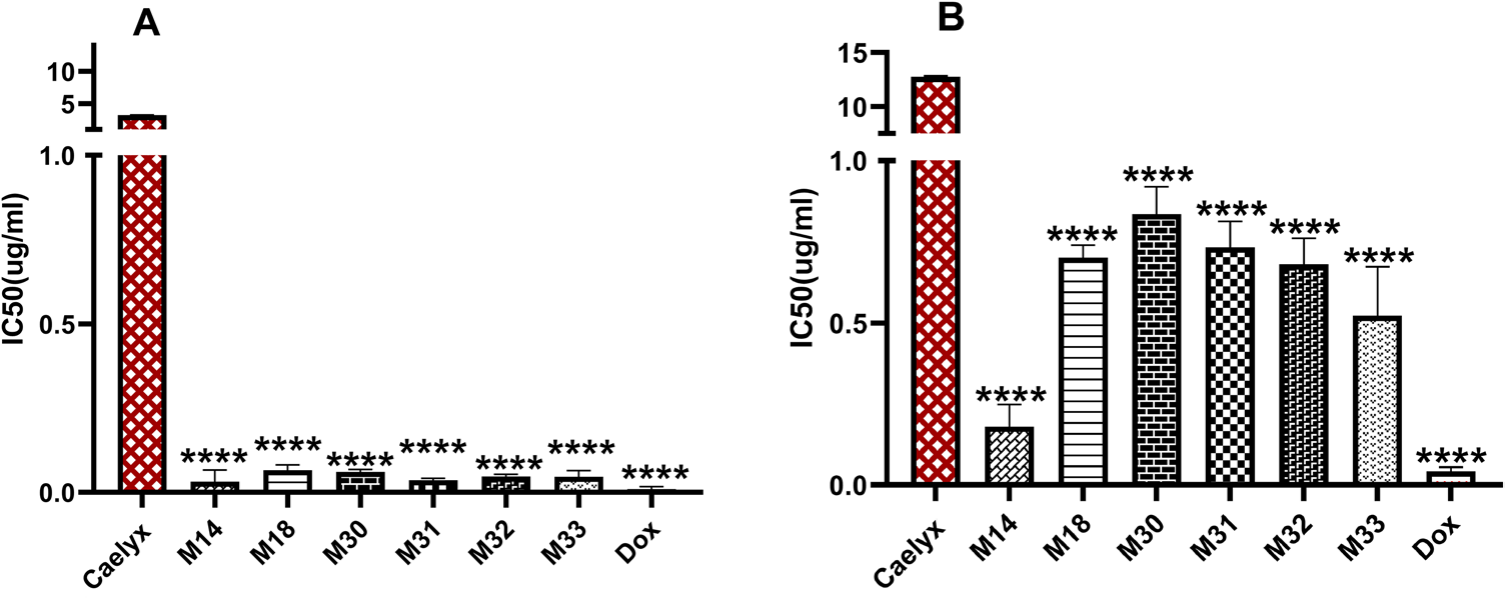
In vitro cytotoxicity of RS liposomal formulations against (**A**) C26 cells, and (**B**) NIH cells. The IC50 was measured by MTT assay following 48 h treatment. The data represented as the means ± SD (n = 3). P value < 0.0001, one-way ANOVA. All formulations are compared to Caelyx.

The cytotoxicity of RS liposomes against C26 and NIH-3T3 cell lines was compared with free Dox (Table 3, Fig. 6A,B). The lowest IC_50_ was observed with M14, which showed the highest in vitro release compared to other formulations. All of the Dox-loaded RS liposomes exhibited higher cytotoxicity against C26 cells compared to NIH-3T3 normal cells at the corresponding concentrations. Dox and Caelyx showed IC_50_ values of 0.01 and 3.24 µg/mL, respectively. The IC_50_ of RS formulations was 0.03–0.06 µg/mL, which was comparable to that of Dox (Fig. 6A). The IC_50_ of RS liposomes against NIH-3T3 cells was in the range of 0.18–0.83 µg/mL, while it was 12.76 µg/mL for Caelyx (Fig. 6B).

**Table 3:** In vitro cytotoxicity of RS liposomes against C26 and NIH cell lines after 48 h treatment.

Formulation	IC50 (C26) 48 h µg/mL	IC50 (NIH) 48 h µg/mL	Ratio of IC50 (NIH)/IC50 (C26)
Dox	0.01 ± 0.010	0.042 ± 0.013	4.20
Caelyx	3.24 ± 0.037	12.76 ± 0.080	3.93
M14	0.032 ± 0.035	0.18 ± 0.069	5.62
M18	0.066 ± 0.016	0.70 ± 0.039	10.60
M30	0.061 ± 0.007	0.83 ± 0.085	13.60
M31	0.036 ± 0.006	0.73 ± 0.080	20.27
M32	0.047 ± 0.007	0.68 ± 0.079	14.46
M33	0.046 ± 0.019	0.52 ± 0.150	11.30

### Doxorubicin cellular uptake

#### Cell uptake using spectrofluorometry

**Figure 7:**
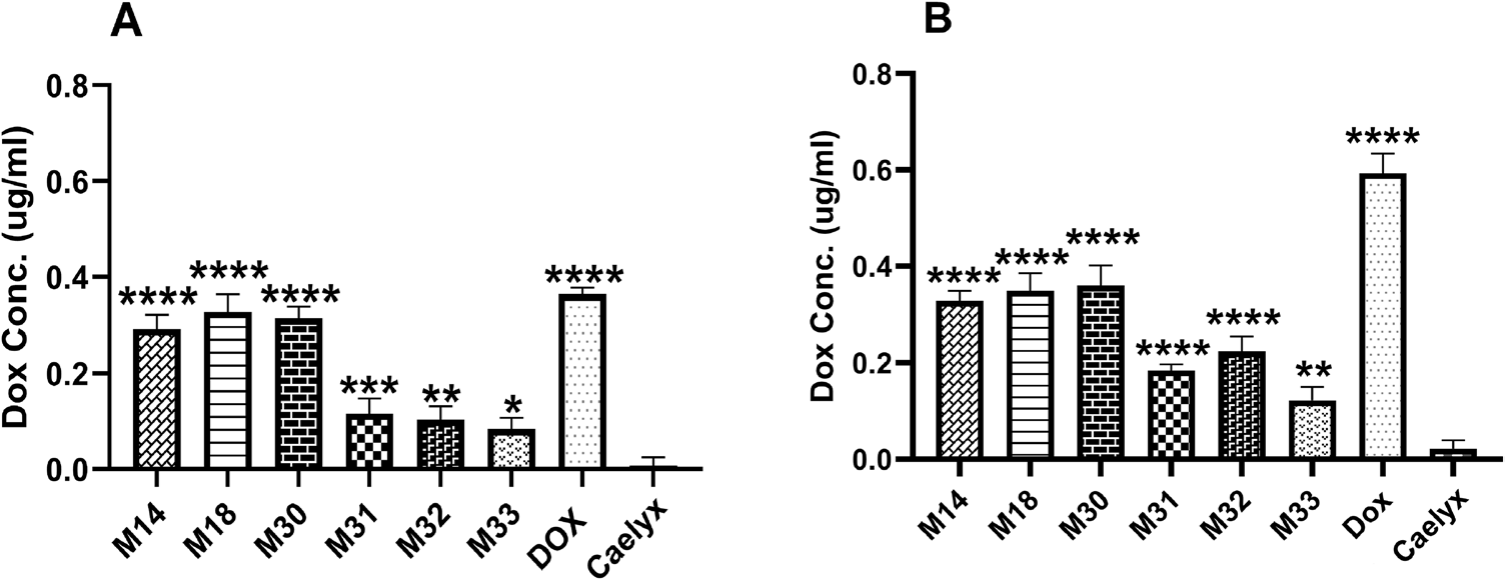
In vitro cellular uptake of free Dox using spectrofluorimetry, Caelyx and RS liposomal formulations after (**A**) 1 h and (**B**) 3 h treat to C26 cells. The data represented as the means ± SD (n = 3). P value < 0.0001, one-way ANOVA. All formulations are compared to Caelyx.

To investigate the Dox intracellular accumulation, RS liposomes were compared to Caelyx after 1 and 3 h incubation. Figure 7, A and B demonstrated the data on cellular uptake, which indicated the significantly enhanced cellular uptake of M14, M18 and M30 following 1 and 3 h (p < 0.001) compared to Caelyx. The highest cellular uptake following 1 and 3 h incubation was observed with free dox.

#### Cell uptake using fluorescence spectroscopy

**Figure 8:**
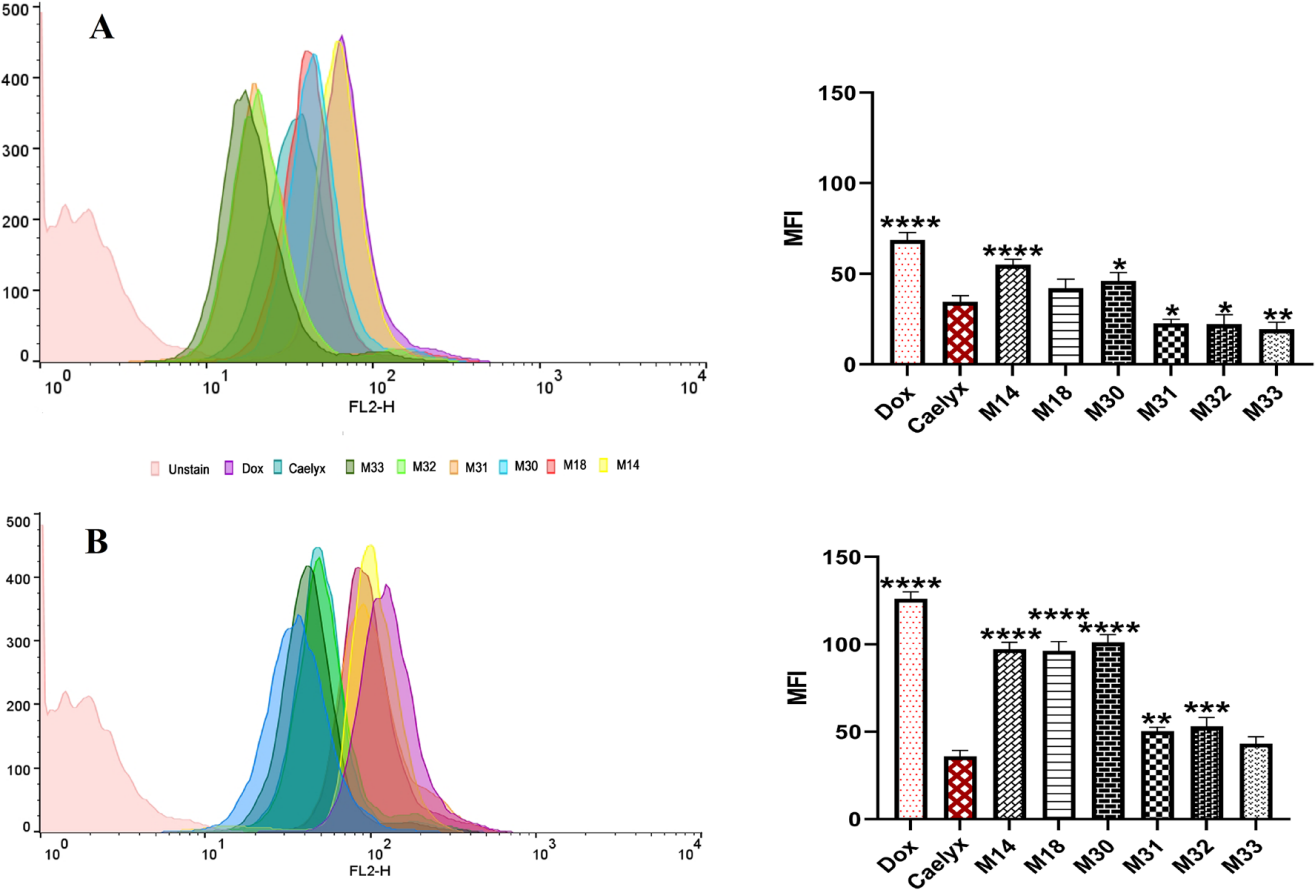
In vitro cellular uptake of free Dox using flowcytometry, Caelyx and RS liposomal formulations after (**A**) 1 h and (**B**) 3 h treat to C26 cells. The data represented as the means ± SD (n = 3). P value < 0.0001, one-way ANOVA. All formulations are compared to Caelyx.

The mean fluorescent intensity (MFI) of the formulations as measured by flow cytometry has shown to be in directly correlate with the amount of Dox internalization based on spectrofluorometry analysis. Figure 8A,B indicated that MFI of C26 cells incubated with either free Dox or various RS liposomes increased over time. Consistent with fluorometry data, M14, M18, and M30 showed the highest MFI compared to all the formulations.

#### Cell uptake using fluorescence microscopy

**Figure 9:**
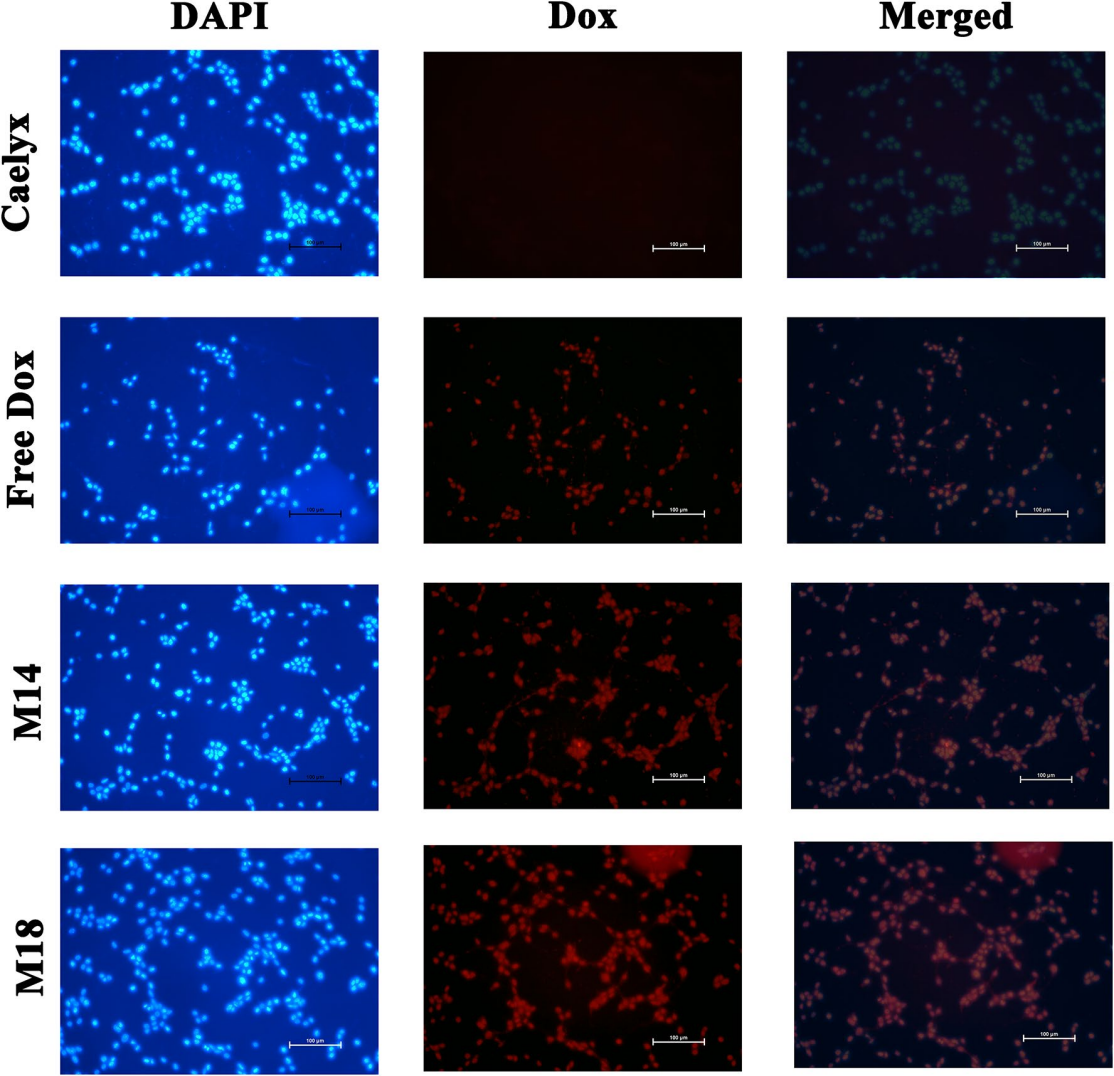
Fluorescence microscopy of doxorubicin on C26 cancer cells. RS liposomal formulations (M14, M18), Caelyx, free Dox (10 µg/mL), DAPI and doxorubicin as well as the merged images are shown after 1 h incubation.

The Dox and RS liposomes uptake in the presence of ROS was evaluated using fluorescence microscopy. As shown in Fig. 9, internalization of the released Dox from M14 and M18 RS formulations bearing DDA bond was more outstanding compared to Caelyx, which was in accordance with spectrofluorometry and fluorescence spectroscopy data.

### In vivo experiments

#### Plasma Dox concentration

**Figure 10:**
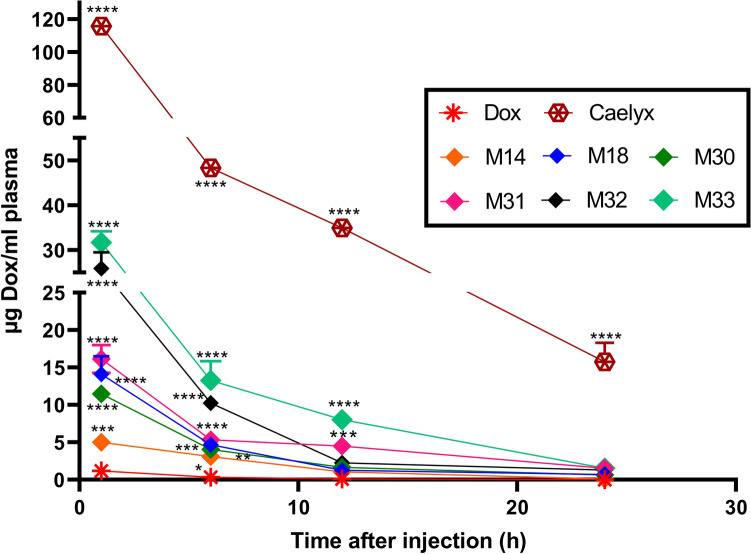
Concentration–time profile of Dox in plasma following administration of free and RS liposomal formulations containing Dox (10 mg/kg). Data are means ± SD (n = 4).

Following i.v. administration, Dox was rapidly eliminated within the first 3.62 h from plasma. The optimum formulations in terms of half-life of Dox were M30 and M31 with the half-lives of 6.93 and 9.60 h, respectively. The mean residence time (MRT) of M30 and M31 were 7.36 and 10.93 h, respectively (Table 4). M32 and M33 bearing 30 and 37.5% of Chol, respectively, exhibited higher concentrations of Dox in the first hours of administration than Chol-free formulations (M14 and M18). Caelyx as the control group, showed the highest concentration of Dox within 24 h (t1/2: 11.03) (Fig. 10). Compared to free Dox, the AUC of RS liposomes significantly increased by approximately 4.6–38.97 fold for M14–M33, respectively (Table 4).

**Table 4:** Pharmacokinetic parameters of Dox following i.v. administration of free and RS liposomal formulations Dox (10 mg/kg).

Parameters	Dox	M14	M18	M30	M31	M32	M33	Caelyx
AUC^a^ (μg/mL h)	7.20	33.16	117.25	88.95	159.10	189.60	280.59	1342.07
MRT^b^ (h)	3.98	4.56	6.00	7.36	10.93	6.39	7.14	13.41
Cl^c^ (mg)/(μg/mL)/h	1.38	0.30	0.08	0.11	0.06	0.05	0.03	0.0074
T_1/2_^d^ (h)	3.62	4.37	5.62	6.93	9.60	5.46	5.41	11.03

^a^Total area under the plasma concentration–time curve.

^b^Mean residence time.

^c^Clearance.

^d^Terminal half-life.

#### Biodistribution study

**Figure 11:**
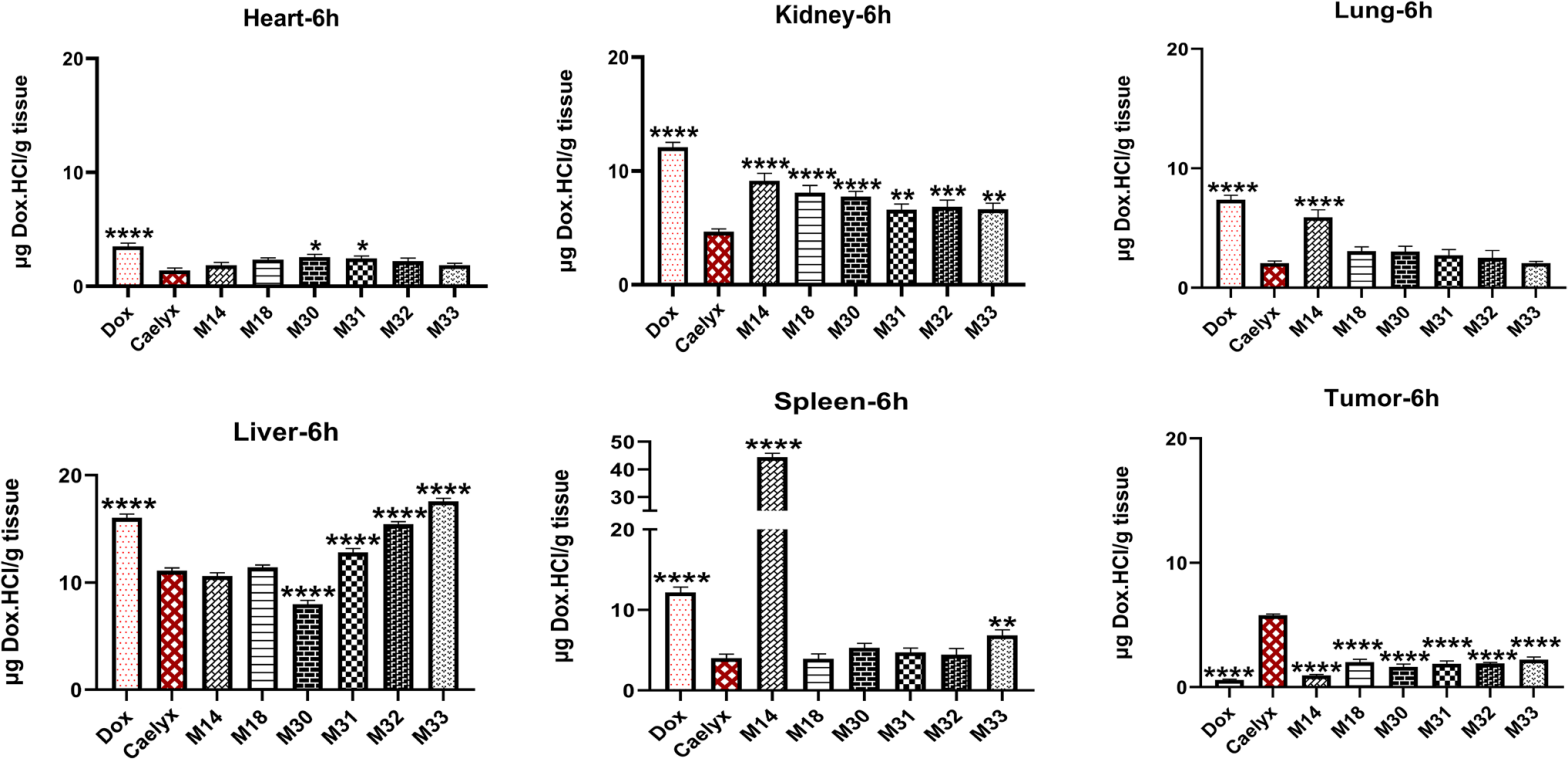
In vivo biodistribution of RS liposomal formulations after 6 h in organs, heart, kidney, lung, liver, spleen and tumor in BALB/c mice bearing C26 tumor after a single dose of 10 mg/kg RS formulations administered i.v. on day 14 after the tumor inoculation (****indicates the P-value ˂ 0.0001). All formulations have been compared to Caelyx.

**Figure 12:**
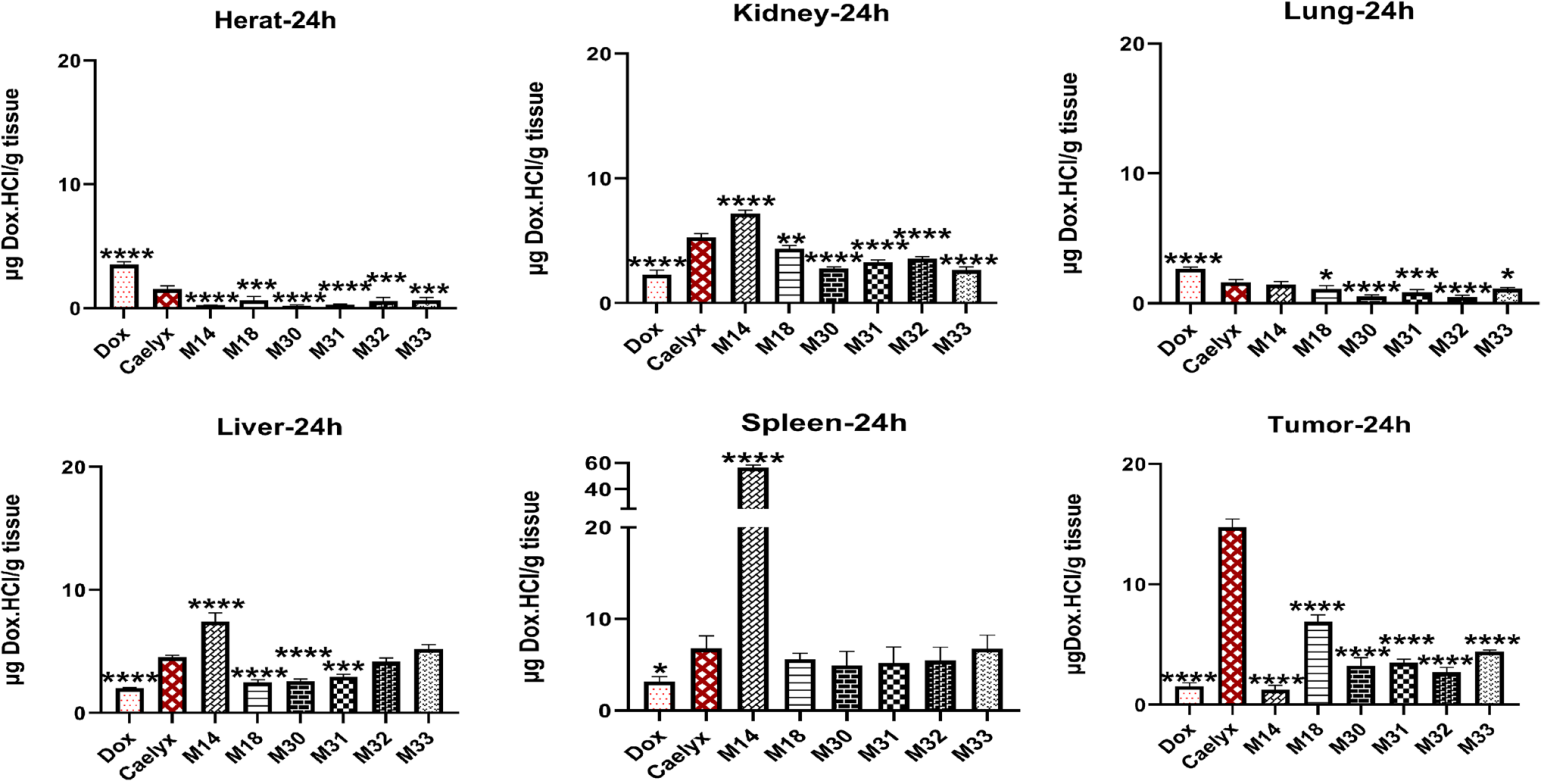
In vivo biodistribution of RS liposomal formulations after 24 h in organs, heart, kidney, lung, liver, spleen and tumor in BALB/c mice bearing C26 tumor after a single dose of 10 mg/kg RS formulations administered i.v. on day 14 after the tumor inoculation (****indicates the P-value ˂ 0.0001). All formulations have been compared to Caelyx.

The in vivo biodistribution of free Dox, Caelyx, and RS liposomes was investigated in mice bearing C26 tumor models after 6 and 24 h (10 mg/kg Dox). As shown in Figs. 11 and 12, after 6 h, the accumulation of Dox in the liver for all RS liposomes was similar to or higher than Caelyx, however, it decreased to less than 5 µg/g of tissue after 24 h, for M18, M30, and M31. Liver Dox concentration of M32 and M33 was similar to that of Caelyx. In the spleen, no significant differences were observed after 6 h between RS formulations and Caelyx except for M14 (at two time points) and M33 (at time point 6 h). The splenic Dox concentration in M14 was significantly greater compared to all other RS liposomes after both 6 and 24 h.

At the tumor site, the amount of Dox after 6 h was almost close in M14–M33 and reached the peak at 24 h for M18. At this time point, the accumulation of Dox for M14 was the least and M30, M31, M32, M33 exhibited almost the same concentrations of Dox (p ˂ 0.0001). Interestingly, Dox concentrations in the heart as the most critical target tissue of the drug was less than 3 µg/g of tissue at time point 6 and less than 1 µg/g of tissue after 24 h (p ˂ 0.0001 compared to Caelyx), for all RS liposomal formulations. Analysis of the lung indicated lower accumulation of Dox following RS liposomes treatment after 6 h, which was significantly decreased for M30, M31, and M33 at 24 h (less than 1 µg/g of tissue) (p ˂ 0.0001 compared to Caelyx). Dox concentrations in the kidney for all RS liposomes were less than 10 µg/g of the tissue at time point 6, (p ˂ 0.0001 compared to Caelyx). After 24 h, the concentration of Dox for M14 was still 7.15 µg/g kidney.

#### Anti-tumor efficacy study

**Figure 13:**
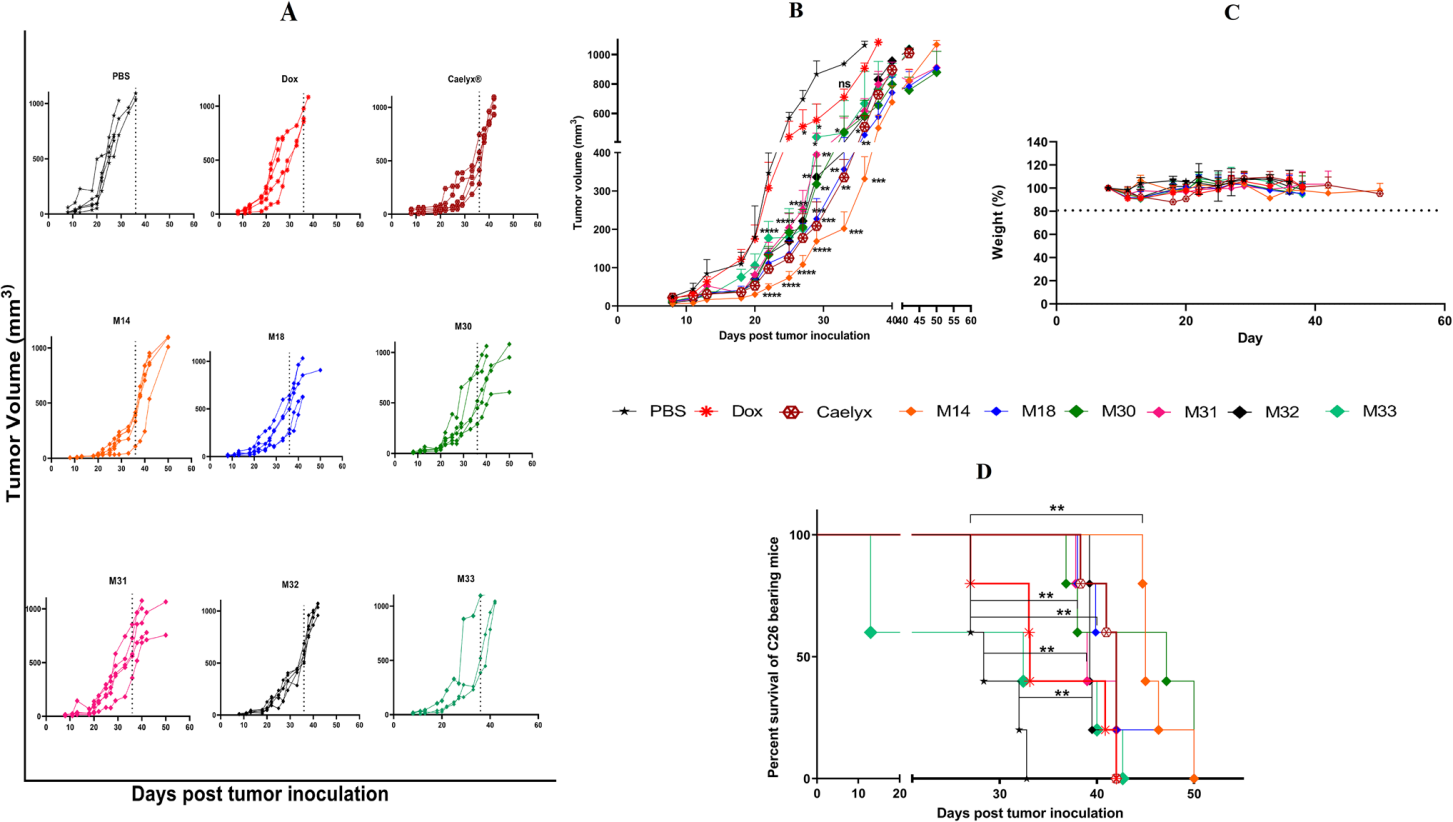
In vivo anti-tumor efficacy of RS liposomal formulations, free Dox, Caelyx and PBS in female BALB/c mice after i.v. administration of a single dose of 10 mg/kg Dox on day 10 after tumor inoculation. (**A**,**B**) Tumor growth curves (**C**) percentage change in animal body weight, and (**D**) survival curve. Data represented as mean ± SD, n = 5; (P-value < 0.0001).

Following i.v. injection of RS liposomes, Caelyx, free Dox, and PBS, anti-tumor efficacy and survival, as well as the changes in the animal’s body weight, were investigated for 50 days. The tumor growth curves represented in Fig. 13, indicated the therapeutic efficacy of all RS liposomes compared to PBS and free Dox groups. Table 5 compared the survival factors including TTE, %TGD, MST and ILS of all treatments. Results indicated that M14 showed the highest TTE of 46.20 ± 2.21 days, followed by M30 and M18 (44.39 ± 6.49, 42.37 ± 4.57 days) respectively, which was even greater compared to Caelyx with the TTE value of 41.06 ± 1.59 days. According to Fig. 13A,B, the monotonous tumor growth rate within mice in one group related to M14 and M32 is observed. Among RS liposomes, M33 showed the lowest TTE value of 28.21 ± 14.39 days which was close to the Dox group (27.59 ± 15.52). The results of animal weight monitoring in Fig. 13C indicated no differences between Caelyx and RS liposome receiving groups. Figure 13D depicts the survival time of groups determined by the Kaplan–Meier method.

**Table 5:** Therapeutic efficacy data of RS liposomal formulations in BALB/c mice bearing C26 colon cancer (p < 0.0001).

Treatments	TTE^a^ (days ± SD)	TGD^b^ (%)	MST^c^ (days)	ILS^d^ (%)
PBS	29.42 ± 2.77	–	28.35	–
Dox.HCl	32.40 ± 5.94	10.12	33.10	13.54
Caelyx	41.06 ± 1.59	39.56	42.00	48.14
M14	46.20 ± 2.21	57.06	45.00	58.73
M18	42.37 ± 4.57	44.03	42.00	48.14
M30	44.39 ± 6.49	59.90	47.15	66.31
M31	41.36 ± 5.11	40.59	39.00	37.56
M32	39.85 ± 1.20	35.47	39.27	38.51
M33	28.21 ± 14.39	− 4.00	32.42	14.35

^a^Time to reach end point.

^b^Tumor growth delay.

^c^Median survival time.

^d^Increase life span.

## Discussion

In the current study, novel redox-sensitive liposomal formulations were developed to improve the Dox release in the presence of ROS, especially H_2_O_2_ overproduced in the TME^32^. To develop liposomal formulations containing DDA, we started from DOPE/Egg PC/DOPC as phospholipids with low Tm and citrate as the hydration buffer. DDA at different ratios was utilized to achieve optimal formulations in terms of stability and without considerable aggregation after drug loading. In situ drug loading using a pH gradient and citrate complex allowed a considerable drug to lipid ratio and high encapsulation efficiency (100%) for RS liposomes^33^.

The organoselenium compound we have synthesized possesses an amphiphilic structure, 18 CH_2_ groups as the nonpolar moiety and carboxylic acid groups as the polar portion of the structure. Due to its amphiphilic structure, DDA could accommodate in both core and bilayer of the liposomes. SP3 configuration of the carbon at the bonding point of Se to CH_2_ could expose the carboxylic acid moieties on the surface of liposomes, or place it inside the hydrophilic core. In the TME, the reactive oxygen species and, more frequently H_2_O_2_, could penetrate the bilayers of the liposomes and, by breaking the diselenide bond, destabilize and collapse the structure of liposomes^34^. It’s been shown that the nonpolar H_2_O_2_ with a mean diameter of about 0.25–0.28 nm^35^ can freely diffuse through the membrane through the aquaporin channels^36^. Here, the optimum RS liposomes contained 2.5% DDA, while the higher DDA ratios (> 2.5%) resulted in the instability of liposomes following hydration and drug loading steps. Due to PEGylation (5%), the aggregation of liposomes in serum was significantly reduced while the blood circulation time increased^37^. Chol has been shown to increase phospholipid packing, Tm, retention time and plasma stability, leading to the overall enhancement of liposomal stability^38^.

Loading of Dox into liposomes was driven by the pH gradient. For this, sodium carbonate added to the aqueous suspension of liposomes created a neutral pH of 7.8 outside the liposomes while the inside pH remained acidic (pH: 4). The formation of Dox complexes with citrate anions inside the vesicles leads to a considerable Dox accumulation (EE: 100%). RS liposomes entrapping citrate exhibited faster loading kinetics, higher loading efficiency, and no aggregation following Dox loading compared to their ammonium sulfate counterparts. The three components M14 formulation (DOPE/Egg PC/DDA) achieved the maximum zeta potential after Dox loading (− 19.50 ± 0.05 mV). Liposome stability evaluation of RS liposomes was done with blank liposomes due to the in situ drug loading method used, similar to Myocet and Thermodox^39–41^. The stability of liposomes during 12 months at 2–8 °C resulted in negligible changes in both size and zeta potential. The increase of the Chol contents in M32 and M33 to 30 and 37.5%, respectively, resulted in the instability and precipitation of DDA after 6 months due to the possible interaction between Chol and DDA. It’s been shown that Chol, due to the functional hydroxyl group, can react with the carboxylic acid of DDA (esterification of Chol), through a chemical reaction called Fischer Esterification^42,43^. Our results indicated the critical role of DOPE in the stability of RS liposomes, since decreasing DOPE ratios in M32 and M33 formulations induced liposomal structure collapse. DOPE, a zwitterionic neutral lipid, when incorporated into liposomal formulations, could increase membrane rigidity similar to Chol, leading to in vitro/in vivo stability of liposomes^44,45^. In our study, by optimizing Chol and DOPE ratios in M30 and M31 formulations (10/27.5 and 20/17.5), we could achieve significant drug-free liposomal stability during 12 months.

In vitro Dox release of RS liposomes increased up to 60% at all tested pH values compared to Caelyx. The underlying reason might be the differences in the internal buffer (citrate) as well as the phospholipids of lower Tm used for the preparation of liposomes. We have also observed that increasing the Chol contents could confer rigidity to the bilayers, resulting in less Dox release from the RS liposomes^46^. This trend was observed in plasma (Fig. 5D), keeping the physiological pH of 7.4. At pH 7.4, M14 showed the maximum Dox release due to the lack of mPEG_2000_–DSPE and Chol in its membrane. At lowered pH (6.5, 5.5), the behavior of Dox release from RS liposomal formulations varied, highlighting the destabilization of the formulations with lower DOPE contents (Fig. 5B,C). To investigate the release pattern in the presence of H_2_O_2,_ we have used RS liposomes containing pyrvinium phosphate as a model, since chromatography results in Supplementary Fig. S1 indicated that Dox was oxidized after 30 min exposure to hydrogen peroxide. RS liposomes containing pyrvinium phosphate showed a burst release (30%) in the presence of 0.1% H_2_O_2_ that increased to 70% during 24 h. The release pattern of RS liposomes showed the successful cleavage of the diselenide bond in all formulations.

Cellular uptake study clearly indicated an enhanced accumulation of RS liposomes. While formulations with lower Chol contents (M14, 18, and 30) showed higher Dox uptake in C26 cells, Chol incorporation at higher ratios augmented the stability of liposomes and lowered drug uptake^45^. These results were in accordance with cytotoxicity tests indicating the greater toxicity of RS liposomes compared to Caelyx. Due to the higher generation of ROS in the cancer cells, the diselenide bond in RS liposomes could be easily cleaved, leading to a higher release of Dox and the subsequent toxicity.

In this study, the pharmacokinetic profiles of Dox in nearly all RS liposomal formulations were improved compared to free Dox. In vivo biodistribution study demonstrated a considerable tumor accumulation of drug following RS liposomes administration after 24 h specifically regarding M18 formulation. It is worth noting that Dox accumulation in the heart, which is the main target of Dox, showed a significant decrease in RS liposomal formulations. The same trend was observed with lung and some extent, with spleen tissues. The higher Dox accumulation of some formulations in the liver and spleen might be attributed to the PEG and Chol contents as well as the size of liposomal formulations. The nanoparticles size of ˃ 200 nm, could explain the greater accumulation within the liver and spleen. Further, the negative surface charge might greatly contribute to the decreased internalization in the tumor site^47^. It seems that RS liposomes could effectively improve TTE and MST survival factors following tumor therapy. In this case, M14, M30, and M18 showed greater efficacy in shrinking the tumor size as compared to Caelyx.

In general, M14 exhibited enhanced anti-tumor efficacy and improved the survival of animals compared to Caelyx. Due to the inclusion of low Tm phospholipids, the absence of cholesterol, and mPEG_2000_–DSPE, as well as the diselenide link cleavage triggered by ROS or glutathione, RS liposomes showed a substantial Dox release in the TME in animals. Further, the colloidal stability of the formulation in circulation and membrane destabilization and hexagonal phase II conversion following exposure to the acidic TME due to the presence of DOPE (37.5%) in the liposome membrane could contribute to the enhanced Dox release within the tumor tissue^17^. There is also evidence indicating that following liver and spleen accumulation, the slow release of liposomal Dox from these tissues over time could enhance the tumor redistribution of the drug through the EPR effect^48^. These features highlight the potential of engineered intelligent liposomes as a novel nanomedicine in the treatment of various malignancies. However, several factors can impose important hurdles limiting the appearance of such systems on the market, irrelevant of whether they are therapeutically beneficial or not. These factors are including large-scale manufacturing, biological challenges, biocompatibility and safety, government regulations, intellectual property, and overall cost-effectiveness in comparison to current therapies.

## Conclusion

The novel Dox redox-sensitive liposomal formulations composed of 10,10′-diselanediylbis decanoic acid (DDA) were successfully prepared and showed significant stability during the 12 months observation. RS liposomal formulations indicated an in vitro burst release of Dox in the presence of H_2_O_2_. In vivo study proved the sufficient Dox release ability of M14 in TME contributed to significant tumor growth suppression and improved survival in the C26 tumor-bearing mouse model. These formulations merit further investigations due to their potential anti-tumor effects.

## Experimental section

### Material

Dioleoylphosphatidylethanolamine (DOPE), Egg l-α-phosphatidylcholine (Egg, Chicken), 1,2-dioleoyl-sn-glycero-3-phosphocholine (DOPC), Methoxy-polyethylene glycol (MW 2000)-distearoylphosphatidylethanoloamine (mPEG2000–DSPE), Cholesterol (Chol), were purchased from Avanti Polar Lipid (Alabaster, USA). Doxorubicin hydrochloride (Dox), Dowex, selenium powder (Se 200 mesh), Sodium borohydride (NaBH_4_), 10-Bromodecanoic acid, magnesium sulfate (MgSO_4_), Hydrogen peroxide (H_2_O_2_) solution 30% (w/w), Dimethylformamide (DMF), dimethyl sulfoxide (DMSO), Diethyl ether, Ethanol 98%, 3-(4,5-dimethylthiazol-2-yl)-2,5-diphenyl tetrazolium bromide (MTT) were obtained from Sigma-Aldrich (Taufkirchen, Germany), C26 cell line (murine colon carcinoma cells) and NIH-3T3 cell line (mouse embryonic fibroblast cells) were purchased from Cell Lines Service (Eppelheim, Germany), RPMI 1640 and DMEM culture media and fetal bovine serum (FBS) were purchased from Gibco (Carlsbad, CA). 4′,6-diamidino-2-phenylindole (DAPI) (Sigma-Aldrich, Taufkirchen, Germany), for nuclear staining. Commercially available Caelyx was purchased from Behestan Darou Company (Tehran, Iran). All other used solvents and reagents were at chemical grade.

### Synthesis of 10,10′-diselanediylbis decanoic acid (DDA)

DDA was prepared according to a previously described method (Fig. 2A)^49^. Briefly, elemental selenium (2 g, 0.025 mol) and NaBH4 (1.9 g, 0.05 mol) were placed in a two-necked round-bottomed flask followed by the addition of 98% EtOH (70 mL). The mixture left stirring under an inert atmosphere at room temperature. The reaction was continued to the end of complete dissolution of selenium and the formation of colorless suspension with white–gray solid. Then, DMF (50 mL) was added to the stirring solution until the color turned red-brown and stirring continued by adding 98% EtOH (25 mL). Following the vigorous stirring and gas evolution, Se powder (200 mesh, 2 g, 0.025 mol) was added to the solution until the Se powder was completely dissolved and a clear dark-red solution appeared. Then, 10-bromodecanoic acid (0.05 mol) was added dropwise to the solution resulting in a color change to yellow. ^1^HNMR of 10-bromodecanoic acid is shown in Fig. 2B. After 24 h, the reaction was quenched by adding distilled water (150 mL). The reaction mixture was then extracted with diethyl ether (100 mL), followed by washing with water (250 mL) three times and drying over MgSO_4_. The crude DDA was obtained after solvent removal using a rotary evaporator. ^1^HNMR, CNMR, and LC–MS/MS techniques were applied to confirm the formation of DDA (Fig. 2C–E). ^1^HNMR results: ^1^H NMR (CDCl_3_, 400 MHz, δ): 1.26 (m, 20H, –CH_2_), 1.57 (m, 4H, *–CH*_*2*_CH_2_Se–), 1.66 (m, 4H, –*CH*_*2*_CH_2_COOH), 2.29 (t, 4H, –*CH*_*2*_Se–), 2.84 (t, 4H, –*CH*_*2*_COOH).

### Preparation of redox-sensitive (RS) liposomes

To develop RS liposomal formulations composed of an amphiphilic organoselenium compound, we started with two-component liposomal formulations including DOPE/Egg PC or DOPC/DOPE at different ratios and using various buffers (Citrate and ammonium sulfate). In the next step, the DDA compound we added to the formulations. Various percentages of DDA ranged from 2.5 to 20%, mPEG_2000_–DSPE (1, 2.5, 5%) and Chol were applied to set up the most stable and well-characterized formulations. Characterization data of all prepared formulations from M1 to M33 are present in the supplementary file (Supplementary Table S1). The formulations of RS liposomes were developed by the lipid film hydration method^50^. Briefly, the mixture of lipids containing DOPE: Egg PC: mPEG_2000_–DSPE: Chol: DDA dissolved in chloroform, was added in a glass tube. The thin lipid film was formed in a rotary evaporator and the trace of chloroform was removed using a freeze-dryer. The lipid film was hydrated in a citrate solution (300 mM) at 58 °C, sonicated for 5 min and was sequentially extruded through polycarbonate membranes of 200, 100, and 50 nm. Sodium bicarbonate was then added to the suspension of nano-sized RS liposomes to make a neutral pH of 7.4 outside the liposomes. Accumulation of Dox into the vesicles was driven by the lower internal pH of RS liposomes (pH: 4). Dox was loaded into liposomes for 10 min at 58 °C. After purification with Dowex resin, the amounts of drug encapsulated in the liposomes were determined by fluorescence spectrophotometer.

### Characterization of liposomes

Particles size, polydispersity index (PDI), and zeta potential were measured by dynamic light scattering (Nano-ZS;Malvern; UK). Phospholipid concentration was measured through Bartlett phosphate assay^51^. To assay doxorubicin concentration, aliquots of Dox loaded RS liposomes were dissolved in acidified isopropyl alcohol below the Dox self-quenching concentration and Dox concentration was measured using spectrofluorimetry (Perkin-Elmer LS-45) (ex: 490 nm/em: 585 nm) through a reference standard curve of serial dilution of Dox^52^. To determine encapsulation efficiency (EE) of Dox, concentrations of Dox were calculated before and after purification. The percent of EE was measured using the following formula:

$$\% {\text{Dox}}\,{\text{encapsulated}} = \, ({\text{Dox}}\,{\text{concentration}}\,{\text{after}}\,{\text{purification}}/{\text{Dox}}\,{\text{concentration}}\,{\text{before}}\,{\text{purification}}) \, \times {1}00.$$

To determine the stability of RS liposomes, they were sealed under argon gas and stored at 2–8 °C. After 3, 6, and 12 months, particle size, zeta potential, and PDI were measured and the appearance of liposomes was visually inspected. To determine the morphological characteristics of liposomes transmission electron microscopy (TEM) (Zeiss, Jena, Germany) was used.

### In vitro release

In vitro release of Dox from RS liposomes and Caelyx was assessed in different pH (7.4, 6.5, 5.5). Briefly, each formulation (0.5 mL) was placed in a dialysis bag (12–14 kDa molecular weight cut-off or MWCO) in a 50 mL phosphate (pH: 7.4 and 6.5) and succinate buffer (pH: 5.5). At defined time intervals (0, 15, 30, 45 and, 60 min, and 2, 4, 6, and 24 h), two mL of the medium was withdrawn and replaced with two ml fresh buffer. Aliquots of the collected samples were analyzed for their Dox content. The cumulative release was calculated using the following equation:

Mt(n) = Vr × Cn + Vs × ƩCm, where Mt(n) is the current cumulative mass of released Dox at time t, n is the number (times) of sampling, Cn is the current concentration of Dox in the medium, ƩCm is the summed total of the previously measured concentrations, Vr is the volume of the medium, and Vs corresponds to the volume of the sample removed for analysis^53^.

### In vitro release in plasma

The equal amount of each formulation (0.5 mL) was placed in a 12–14 kDa MWCO dialysis bag containing 50% plasma in a 50 mL medium of dextrose. At determined time points of 0, 15, 30, 45, 60 min, and 2, 4, 6, and 24 h, 2 mL of the medium was withdrawn and replaced with 2 mL fresh buffer. Aliquots of the collected samples were analyzed for their Dox content. The cumulative release was calculated using the above-mentioned equation.

### In vitro release in the presence of 0.1% H_2_O_2_

Due to the oxidation of Dox in the presence of H_2_O_2_, a model of RS liposomal formulations containing pyrvinium phosphate was selected to investigate the sensitivity of liposomes to ROS and investigation the release of Dox.

#### Preparation of pyrvinium phosphate

Pyrvinium phosphate was previously synthesized in our laboratory^54^. Briefly, a sample of pyrvinium pamoate, 0.51 g, was placed in a 250 mL Erlenmeyer flask with a magnetic stirring bar. Then, 40 mL chloroform was added, followed by adding 20 mL of 95% ethanol, resulting in a deep red-colored solution. The mixture heated to 50 ℃ and stirred for 10 min and then was precipitated following the addition of 10 mL of 2% phosphoric acid (85%) in 95% ethanol. After 2 min, 30 mL of ethyl acetate was added, and the mixture was stirred for another 20 min. The solids were then collected by filtration, washed with a 20 mL mixture of ethyl acetate:chloroform:ethanol (2:1:1), and air-dried to provide 0.45 g of brick-red powder. A sample of the phosphate salt was soluble in water at 1 mg/mL, giving an orange-red colored solution. This contrasts the poor solubility of pyrvinium pamoate with the water solubility of 0.000288 mg/mL^55^.

#### Preparation of RS liposomal formulations containing pyrvinium phosphate

Thin films of DOPE: Egg PC: DDA: mPEG_2000_–DSPE: Chol at different molar ratios were prepared and hydrated with PBS containing 1 mg/mL pyrvinium phosphate at 58 °C. After extrusion, sonication, and dialysis, the concentration of pyrvinium phosphate was determined using a spectrofluorometer (Shimadzu RF5000U, Japan) (ex: 560 nm/em: 680 nm).

#### Release study in the presence of 0.1% H_2_O_2_

RS liposomes containing pyrvinium phosphate were placed in a dialysis bag (12–14 kDa MWCO) in a 50 mL phosphate buffer (pH: 7.4, 6.5) containing 0.1% H_2_O_2_. At determined time points (0, 15, 30, 45, and 60 min, and 2, 4, 6, and 24 h), 2 mL of buffer was removed and replaced with 2 mL fresh buffer. All samples were collected and analyzed for pyrvinium phosphate contents at ex: 560 nm/em: 680 nm. The cumulative release was calculated using the following equation as previously mentioned.

$${\text{Mt}}\left( {\text{n}} \right)\, = \,{\text{Vr }} \times {\text{ Cn}}\, + \,{\text{Vs }} \times \, \sum {{\text{Cm}}} ,$$

### Cell culture

C26 and NIH-3T3 cell lines were cultured at 37 °C in a 5% CO2/95% air humidified atmosphere in RPMI 1640 and DEMEM media, respectively; the media were supplemented with 10% FCS and 100 U/mL penicillin, and 100 μg/mL streptomycin.

### Cytotoxicity study

The cytotoxicity of RS liposomal Dox was evaluated using MTT assay. C26 and NIH-3T3 cells were seeded at 5000 cells/well into 96-well plates. After overnight incubation, cells were treated with different concentrations of free Dox, Caelyx, and RS liposomal Dox. After 48 h incubation at 37 °C, the cells were washed with PBS and MTT (10 μL/well MTT and 100 μL/well cell media) was added. After an additional incubation for 4 h at 37 °C, the absorbance of MTT was measured using a microplate reader (Awareness Technology Inc., USA) at 570 nm. Relative cell death (R) was calculated as follows:

R = 1 − [(A sample − A blank)/(A control − A blank)], where A sample and A control are the absorbances of the cells incubated with the sample solutions and the culture medium as the control group, respectively. A blank is the absorbance of MTT solution in a well without cells. The IC50 values were calculated using CalcuSyn Version 2.0 software (BIOSOFT, UK)^52^.

### Doxorubicin cellular uptake

#### Cell uptake using fluorescence spectroscopy

C26 cells were seeded at 1 × 10^6^ cells/well in 6-well plates and incubated overnight. Then the medium was replaced with FCS free medium containing 10 mg/mL of Caelyx, RS liposomal Dox, and free Dox. After 1 and 3 h of incubation at 37 °C, cells were washed with cold PBS. Then, acidified isopropyl alcohol (0.9 mL) was added to each well and transferred to a 2 mL vial and stored at 4 °C for 24 h. Samples were centrifuged for 10 min at 14,000 rpm. The supernatant was then collected and Dox concentration was calculated after preparation of serial dilutions of Dox^56^.

#### Cell uptake using flow cytometry

Cellular uptake was quantitated using flow cytometry to confirm the results of spectrofluorimetry. Cells were seeded into 24-well plates at a density of 1 × 10^5^ cells/well in 2 mL media with 10% FBS. After overnight incubation, the medium was replaced with 1 mL FBS free medium containing formulations including Caelyx, RS liposomes, and free Dox with a final drug concentration of 10 μg/mL, and incubated at 37 °C for 1 and 3 h. Cells cultured with media were regarded as controls. Following incubation, cells were washed three times with cold PBS and were detached by trypsin-EDTA solution. Then, the cell washing process was done through centrifuging at 1500 rpm for 5 min and re-suspending in 1 mL PBS containing 3% FBS. After three times washing, cells were suspended in 0.3 mL PBS containing 3% FBS and subjected to flow cytometry analysis using FlowJo Software version 10 (FlowJo, Ashland, US)^57^.

#### Evaluation of intracellular Dox uptake using fluorescence microscopy

Fluorescence microscopy analysis was used to detect the effect of ROS on diselenide bond cleavage in RS formulations and the subsequent Dox internalization into cancer cells. For this, sterile coverslips were placed at each well of a 6-well plate and 5 × 10^5^ C26 cells were seeded in each well and incubated overnight at 37 °C with 95% air and 5% CO_2_. Then, M14, M18, Caelyx, and free Dox (10 µg/mL Dox) were added to each well and incubated for 1 h. Cells were then washed three times with PBS and fixed with 4% paraformaldehyde (10 min). After washing again with PBS, cells were incubated with DAPI labeling solution in the dark and the coverslips were mounted on the microscopic slide. Intrinsic fluorescent of Dox assisted to evaluate drug cell uptake using Olympus BX-51 fluorescence microscope (Olympus, Japan)^58,59^.

### Plasma elimination of Dox loaded RS liposomal formulations

BALB/c mice (aged 6–8 weeks, 18–20 g) were purchased from the Pasteur Institute (Tehran, Iran). All animal experiments were approved by the Institutional Ethical Committee and Research Advisory Committee of Mashhad University of Medical Sciences guidelines (Ethical number: 960881) and all animal experiments and methods were carried out in accordance with the relevant guidelines and regulations approved by the ethical committee and ARRIVE guidelines^60^. Furthermore, according to the Guide for the Care and Use of Laboratory Animals, mice were euthanized when they met the euthanasia criteria, including dramatic body weight loss (> 20% of initial weight), tumor volume of > 1000 mm^3^, or inability to feed. The mice were kept in an animal house of Pharmaceutical Research Center in a colony room with a cycle of 12/12 h light/dark at 21 °C with free access to animal food and water. BALB/c mice received a single bolus tail vein injection of RS liposomes, Caelyx, and free Dox (10 mg/mL Dox). At different time points, 300 μL of blood were collected by retro-orbital puncture into EDTA containing tubes and were immediately centrifuged at room temperature for 10 min (4000×*g*) to obtain plasma. The resulting plasma samples were diluted with acidified isopropyl alcohol and were stored at 4 °C for 24 h. Afterward, samples were centrifuged at 4 °C for 20 min (4000×*g*) and the Dox concentration was measured after preparation of a series of Dox standards using a spectrofluorometer (Perkin-Elmer LS-45) (ex: 490 nm/em: 585 nm)^61^.

### Pharmacokinetic study

The pharmacokinetic parameters were calculated using the Microsoft Excel add-in program PK Solver. The non-compartmental model was chosen to perform the pharmacokinetic analysis. This included the total area under the plasma concentration–time curve from time zero to time infinity (AUC), mean residence time (MRT), time-averaged total body clearance (CL), and terminal half-life (t_1/2_).

### Biodistribution study

BALB/c mice aged 4–6 weeks were injected subcutaneously by C26 tumor cells (350,000 cells per mouse) in the right flank. Roughly, two weeks later, when the tumor size reached 5 mm diameter, mice were randomly divided into 9 groups (n = 6). Mice were injected via the tail vein with 10 mg/kg of Dox, Caelyx, RS liposomes, and PBS as control. At 6 and 24 h post-injection, mice were sacrificed (3 mice at each time). The whole tumor tissues, a portion of livers, spleens, hearts, kidneys, and lungs were dissected, weighed and homogenized in 1 mL of acidified isopropanol in tubes containing zirconia beads (Mini-Beadbeater-1 Biospec, UK). All samples were stored at 4 °C overnight to extract doxorubicin. After 24 h, all samples were centrifuged and Dox concentration in supernatants was assayed using a spectrofluorometer (ex: em, 490:585 nm). The calibration curves were prepared using serial dilutions of Dox in the tissues of the control mice^62^.

### Therapeutic efficacy

Tumor inoculation was conducted as mentioned in the biodistribution section before. Tumors were then allowed to grow until inoculated mice had palpable tumors (8 days). Mice were then randomly divided into 9 groups (n = 5), including RS liposomes, Caelyx, free Dox, and control group. All groups except the control group received 10 mg/kg Dox via single vein tail injection. Control mice received 200 µL of PBS. From the first day of treatment, the animals’ weight, tumor volume, and overall health were monitored three times a week for 60 days. Tumor volume was calculated with calipers in three dimensions using the following formula:

Tumor volume (mm^3^) = (height × length × width) × 0.5.

For ethical consideration, the exclusion criteria were: (1) tumor enlargement (more than 2 cm in one dimension), (2) bodyweight loss of more than 15% of the initial mass, (3) to become sick or lethargic. The survival results were determined by Kaplan–Meier analysis. The time to reach the end-point (TTE) for each mouse was calculated from the line equation obtained by logarithmic regression of the tumor growth curve. The percent of tumor growth delay (%TGD) was calculated from the following formula^63–65^:

$$\% {\text{TGD}}\, = \,[\left( {{\text{mean}}\,{\text{TTE}}\,{\text{of}}\,{\text{treatment}}\,{\text{group}}{-}{\text{mean}}\,{\text{TTE}}\,{\text{of}}\,{\text{control}}\,{\text{group}}} \right)/{\text{mean}}\,{\text{TTE}}\,{\text{of}}\,{\text{control}}\,{\text{group}}]\, \times \,{1}00.$$

### Statistical analysis

Statistical analysis was performed using GraphPad Prism version 8 (GraphPad Software, San Diego, CA). Survival data were analyzed by the log-rank test. A one-way ANOVA statistical test was used to assess the significance of the differences among various groups for MTT, uptake biodistribution tests. A two-way ANOVA statistical test was used to assess the significance of the differences among various groups for release studies, plasma Dox.conc. evaluation, animal weight and tumor growth. A p-value of > 0.05 was considered statistically significant in all cases.

## Supplementary Information

Supplementary Information.

## Data Availability

The datasets generated during and/or analyzed during the current study are available from the corresponding author on reasonable request.

## References

[1] Chatterjee K (2010). Doxorubicin cardiomyopathy. Cardiology.

[2] Ngan YH, Gupta M (2016). A comparison between liposomal and nonliposomal formulations of doxorubicin in the treatment of cancer: An updated review. Arch. Pharm. Pract..

[3] Rahman AM, Yusuf SW, Ewer MS (2007). Anthracycline-induced cardiotoxicity and the cardiac-sparing effect of liposomal formulation. Int. J. Nanomed..

[4] Lee CC (2006). A single dose of doxorubicin-functionalized bow-tie dendrimer cures mice bearing C-26 colon carcinomas. Proc. Natl. Acad. Sci..

[5] Zhao N, Woodle MC, Mixson AJ (2018). Advances in delivery systems for doxorubicin. J. Nanomed. Nanotechnol..

[6] Andriyanov AV (2014). Therapeutic efficacy of combining pegylated liposomal doxorubicin and radiofrequency (RF) ablation: Comparison between slow-drug-releasing, non-thermosensitive and fast-drug-releasing, thermosensitive nano-liposomes. PLoS One.

[7] Zhao Y (2013). A simple way to enhance Doxil^®^ therapy: Drug release from liposomes at the tumor site by amphiphilic block copolymer. J. Control. Release.

[8] Torchilin, V. P. Passive and active drug targeting: Drug delivery to tumors as an example. *Drug Deliv.* (197), 3–53 (2010). 10.1007/978-3-642-00477-3_1 20217525

[9] Barenholz YC (2012). Doxil^®^—The first FDA-approved nano-drug: Lessons learned. J. Control. Release.

[10] Avnir Y (2011). Fabrication principles and their contribution to the superior in vivo therapeutic efficacy of nano-liposomes remote loaded with glucocorticoids. PLoS One.

[11] Moussa M (2013). Adjuvant liposomal doxorubicin markedly affects radiofrequency ablation-induced effects on periablational microvasculature. J. Vasc. Interv. Radiol..

[12] Gabizon A, Martin F (1997). Polyethylene glycol-coated (pegylated) liposomal doxorubicin. Drugs.

[13] Kashkooli FM, Soltani M, Souri M (2020). Controlled anti-cancer drug release through advanced nano-drug delivery systems: Static and dynamic targeting strategies. J. Control. Release.

[14] Pham SH, Choi Y, Choi J (2020). Stimuli-responsive nanomaterials for application in antitumor therapy and drug delivery. Pharmaceutics.

[15] Li F (2020). Stimuli-responsive nano-assemblies for remotely controlled drug delivery. J. Control. Release.

[16] Qiao Y (2019). Stimuli-responsive nanotherapeutics for precision drug delivery and cancer therapy. Wiley Interdiscip. Rev. Nanomed. Nanobiotechnol..

[17] Lee Y, Thompson D (2017). Stimuli-responsive liposomes for drug delivery. Wiley Interdiscip. Rev. Nanomed. Nanobiotechnol..

[18] Mirhadi E (2020). Redox-sensitive nanoscale drug delivery systems for cancer treatment. Int. J. Pharm..

[19] McCarley RL (2012). Redox-responsive delivery systems. Annu. Rev. Anal. Chem..

[20] Magnani F, Mattevi A (2019). Structure and mechanisms of ROS generation by NADPH oxidases. Curr. Opin. Struct. Biol..

[21] Winterbourn CC (2020). Biological chemistry of superoxide radicals. ChemTexts.

[22] Patel, R. *et al.* Reactive oxygen species: The good and the bad. In *Reactive Oxygen Species (ROS) in Living Cells* (2018). 10.5772/intechopen.71547

[23] Bayr H (2005). Reactive oxygen species. Crit. Care Med..

[24] Weinberg F, Ramnath N, Nagrath D (2019). Reactive oxygen species in the tumor microenvironment: An overview. Cancers.

[25] Tan HW (2018). Selenium species: Current status and potentials in cancer prevention and therapy. Int. J. Mol. Sci..

[26] Cao S, Durrani FA, Rustum YM (2004). Selective modulation of the therapeutic efficacy of anticancer drugs by selenium containing compounds against human tumor xenografts. Clin. Cancer Res..

[27] Ma N (2010). Dual redox responsive assemblies formed from diselenide block copolymers. J. Am. Chem. Soc..

[28] Behroozi F (2018). Smart liposomal drug delivery for treatment of oxidative stress model in human embryonic stem cell-derived retinal pigment epithelial cells. Int. J. Pharm..

[29] Zeng X (2015). Redox poly (ethylene glycol)-b-poly (l-lactide) micelles containing diselenide bonds for effective drug delivery. J. Mater. Sci. Mater. Med..

[30] Liu J (2012). Hyperbranched polydiselenide as a self assembling broad spectrum anticancer agent. Biomaterials.

[31] Wang L (2014). Dual redox responsive coassemblies of diselenide-containing block copolymers and polymer lipids. Langmuir.

[32] Liu J (2013). Therapeutic nanocarriers with hydrogen peroxide-triggered drug release for cancer treatment. Biomacromol.

[33] Swenson C (2001). Liposome technology and the development of Myocet™(liposomal doxorubicin citrate). Breast.

[34] Kim KS (2015). Reactive oxygen species-activated nanomaterials as theranostic agents. Nanomedicine.

[35] Bienert GP, Schjoerring JK, Jahn TP (2006). Membrane transport of hydrogen peroxide. Biochim. Biophys. Acta BBA Biomembr..

[36] Bienert GP (2007). Specific aquaporins facilitate the diffusion of hydrogen peroxide across membranes. J. Biol. Chem..

[37] Xu L, Wempe MF, Anchordoquy TJ (2011). The effect of cholesterol domains on PEGylated liposomal gene delivery in vitro. Ther. Deliv..

[38] Nakhaei P (2021). Liposomes: Structure, biomedical applications, and stability parameters with emphasis on cholesterol. Front. Bioeng. Biotechnol..

[39] Fritze A (2006). Remote loading of doxorubicin into liposomes driven by a transmembrane phosphate gradient. Biochim. Biophys. Acta BBA Biomembr..

[40] Chen K-J (2013). A thermoresponsive bubble-generating liposomal system for triggering localized extracellular drug delivery. ACS Nano.

[41] Bi H (2019). Current developments in drug delivery with thermosensitive liposomes. Asian J. Pharm. Sci..

[42] Carlile SI (1986). Age-related changes in the rate of esterification of plasma cholesterol in Fischer-344 rats. Mech. Ageing Dev..

[43] Joseph T, Sahoo S, Halligudi S (2005). Brönsted acidic ionic liquids: A green, efficient and reusable catalyst system and reaction medium for Fischer esterification. J. Mol. Catal. A Chem..

[44] Chang S-F (2018). The impact of lipid types and liposomal formulations on osteoblast adiposity and mineralization. Molecules.

[45] Tseng LP (2007). Liposomes incorporated with cholesterol for drug release triggered by magnetic field. J. Med. Biol. Eng..

[46] García MC (2021). Cholesterol levels affect the performance of AuNPs-decorated thermo-sensitive liposomes as nanocarriers for controlled doxorubicin delivery. Pharmaceutics.

[47] Di J (2020). Size, shape, charge and “stealthy” surface: Carrier properties affect the drug circulation time in vivo. Asian J. Pharm. Sci..

[48] Blanco E, Shen H, Ferrari M (2015). Principles of nanoparticle design for overcoming biological barriers to drug delivery. Nat. Biotechnol..

[49] Rafique J (2015). Synthesis and biological evaluation of 2-picolylamide-based diselenides with non-bonded interactions. Molecules.

[50] Zhang H (2017). Thin-film hydration followed by extrusion method for liposome preparation. Liposomes.

[51] Bartlett GR (1959). Phosphorus assay in column chromatography. J. Biol. Chem..

[52] Mashreghi M (2021). Improving anti-tumour efficacy of PEGylated liposomal doxorubicin by dual targeting of tumour cells and tumour endothelial cells using anti-p32 CGKRK peptide. J. Drug Target..

[53] Jaafari MR (2009). Effect of topical liposomes containing paromomycin sulfate in the course of Leishmania major infection in susceptible BALB/c mice. Antimicrob. Agents Chemother..

[54] Hatamipour M (2021). Anti-tumor efficacy of pyrvinium pamoate nanoliposomes in an experimental model of melanoma. Anti-Cancer Agents Med. Chem..

[55] Yu D-H (2008). Pyrvinium targets the unfolded protein response to hypoglycemia and its anti-tumor activity is enhanced by combination therapy. PLoS One.

[56] Arabi L (2015). Targeting CD44 expressing cancer cells with anti-CD44 monoclonal antibody improves cellular uptake and antitumor efficacy of liposomal doxorubicin. J. Control. Release.

[57] Horowitz AT, Barenholz Y, Gabizon AA (1992). In vitro cytotoxicity of liposome-encapsulated doxorubicin: Dependence on liposome composition and drug release. Biochim. Biophys. Acta BBA Biomembr..

[58] Mashreghi M (2020). Anti-Epcam aptamer (Syl3c)-functionalized liposome for targeted delivery of doxorubicin: In vitro and in vivo antitumor studies in mice bearing C26 colon carcinoma. Nanoscale Res. Lett..

[59] Chacko SM (2015). Protective effect of p-coumaric acid against doxorubicin induced toxicity in H9c2 cardiomyoblast cell lines. Toxicol. Rep..

[60] Percie du Sert N (2020). The ARRIVE guidelines 2.0: Updated guidelines for reporting animal research. J. Cereb. Blood Flow Metab..

[61] Chi Y (2017). Redox-sensitive and hyaluronic acid functionalized liposomes for cytoplasmic drug delivery to osteosarcoma in animal models. J. Control. Release.

[62] Moosavian SA (2016). Improvement in the drug delivery and anti-tumor efficacy of PEGylated liposomal doxorubicin by targeting RNA aptamers in mice bearing breast tumor model. Colloids Surf. B.

[63] Amin M, Badiee A, Jaafari MR (2013). Improvement of pharmacokinetic and antitumor activity of PEGylated liposomal doxorubicin by targeting with N-methylated cyclic RGD peptide in mice bearing C-26 colon carcinomas. Int. J. Pharm..

[64] Schluep T (2006). Preclinical efficacy of the camptothecin-polymer conjugate IT-101 in multiple cancer models. Clin. Cancer Res..

[65] Zamani P (2020). Nanoliposomal vaccine containing long multi-epitope peptide E75-AE36 pulsed PADRE-induced effective immune response in mice TUBO model of breast cancer. Eur. J. Cancer.

