# Supplementary material for: Redox-sensitive doxorubicin liposome: a formulation approach for targeted tumor therapy
Source: Sci Rep. 2022 Jul 4;12:11310. doi: 10.1038/s41598-022-15239-x (PMC9253031; doi:10.1038/s41598-022-15239-x)
Supplement: Supplementary file 1 — Supplementary Information. [file 41598_2022_15239_MOESM1_ESM.docx]

**Redox-sensitive doxorubicin liposome: a novel formulation approach for improved targeted tumor therapy**

Elaheh Mirhadi MSc ^1^, Mohammad Mashreghi PhD ^1^,

^1^ Nanotechnology Research Center, Pharmaceutical Technology Institute, Mashhad University of Medical Sciences, Mashhad, Iran

^2^ Department of Pharmaceutical Nanotechnology, School of Pharmacy, Mashhad University of Medical Sciences, Mashhad, Iran

*Corresponding author.

Mahmoud Reza Jaafari,

Mashhad University of Medical Sciences, Mashhad, Iran

Tel.: +98 513 1801336;

Fax: +98 513 8823251.

E-mail addresses: jafarimr@mums.ac.ir & [jaafari42@yahoo.com](mailto:jaafari42@yahoo.com)

1. **Preparation of redox sensitive (RS) liposomes**

To develop RS liposomal formulations composed of amphiphilic organoselenium compound we started with two-component liposomal formulations including DOPE/Egg PC or DOPC/DOPE in different ratio and buffers (Citrate and ammonium sulfate). In the next step after selection of the best formulation, we added the diselenide to the formulations. Various percentages of diselenide ranged 2.5-20%, mPEG-DSPE2000 and Chol were applied to set up the the most stable and well-characterized formulations. The formulations of RS liposomes were developed by the lipid film hydration method [13] with various molar ratios of lipids. Briefly, mixture of lipids containing DOPE: Egg PC: mPEG-DSPE2000: Chol: diselenide, dissolved in chloroform, were added in a glass tube. Thin film was formed in a rotary evaporator and the trace of chloroform was removed using freeze-dryer. The lipid film was hydrated in a citrate solution (300 mM) at 58 ºC, sonicated for 5 min and then sequentially extruded through polycarbonate membranes of 200 nm, 100 nm, and 50 nm. Sodium carbonate was added to the suspension of nano-sized RS liposomes to make a neutral pH of 7.4 outside the liposomes (Figure 2). Accumulation of Dox into the vesicles was driven by the lower internal pH of RS liposomes (pH: 4). Dox was loaded into liposomes for 10 min at 58 ºC.

**Table S1.** The physicochemical properties of different RS liposomes

| Name | Formulation | PDI^a^ ± SD^b^ | Z-average ± SD | Intensity ± SD | Volume ± SD | | Number ± SD | Zeta Potential ± SD |
| --- | --- | --- | --- | --- | --- | --- | --- | --- |
| M1 | DOPE/Egg PC 50/50 | 0.509 ± 0.004 | 542.40 ± 6.001 | 720.80 ± 18.56 | 273.30 ± 23.78 | | -20.20 ± 21.96 | -20.20 ± 1.80 |
| M2 | DOPE/Egg PC 60/40 | NH | - | - | - | | - | - |
| M3 | DOPC/DOPE 40/60 | 0.42 ± 0.03 | 1538 ± 4.58 | 660.50 ± 25.30 | 681.2 ± 18.20 | | 657.60 ± 19.40 | -10.7 ± 5.3 |
| M4 | DOPC/DOPE 50/50 | 0.65 ± 0.015 | 406.50 ±n 1.46 | 380.60 ± 12.36 | 399.20 ± 15.75 | | 326.80 ± 16.55 | -9.69 ± 6.15 |
| M5 | DOPC/DOPE 60/40 | 0.464 ± 0.032 | 2874 ± 32.64 | 2116 ± 27.41 | 2043 ± 29.71 | | 1943 ± 25.67 | -5.62 ± 0.36 |
| M6 | HSPC/Chol/mPEG_2000_-DSPE  57/38/5 | 0.242 ± 0.28 | 158.80 ± 14.59 | 164.30 ± 22.73 | 149.7 ± 18.64 | | 104.80 ± 21.26 | -6.57 ± 0.89 |
| M7 | DOPE/Egg PC 40/60 | 0.193 ± 0.041 | 133.00 ± 9.32 | 140.80 ± 23.4 | 123.20 ± 24.21 | | 91.49 ± 14.00 | -4.89 ± 0.24 |
| M8 | DOPE/Egg PC 40/60 | 0.182 ± 0.032 | 128.40 ± 7.45 | 98.00 ± 23.50 | 118.90 ± 24.50 | | 89.40 ± 14.50 | -5.32 ± 1.21 |
| M9 | DOPE/Egg PC/ DDA  40/55/5 | 0.556 ± 0.068 | 242.30 ± 3.85 | 143.70 ± 17.80 | 135.40 ± 19.63 | | 113.13 ± 19.60 | -27.30 ± 2.50 |
| M10 | DOPE/Egg PC/ DDA  40/50/10 | 0.575 ± 0.096 | 524.70 ± 5.64 | 631.10 ± 37.80 | 694.60 ± 41.70 | | 599.20 ± 35.50 | -40.9 ± 2.36 |
| M11 | DOPE/Egg PC/ DDA  40/45/15 | NH**^c^** | - | - | - | | - | - |
| M12 | DOPE/Egg PC/ DDA  40/40/20 | NH | - | - | - | | - | - |
| M13 | DOPE/Egg PC/ DDA  30/50/20 | NH | - | - | - | | - | - |
| M14 | DOPE/Egg PC/ DDA  37.5/60/2.5 | 0.231 ± 0.04 | 223.20 ± 6.57 | 262.50 ± 7.28 | 286.50 ± 6.14 | | 141.43 ± 5.43 | -19.50 ± 0.05 |
| M15 | DOPE/Egg PC/ DDA  35/60/5 | 0.686 ± 0.10 | 178.20 ± 10.54 | 166.20 ± 12.69 | 165.90 ± 15.74 | | 154.00 ± 14.58 | -24.50 ± 1.88 |
| M16 | DOPE/Egg PC/ DDA  32.5/60/7.5 | 0.341 ± 0.082 | 291.10 ± 8.49 | 234.04 ± 18.32 | 220.46 ± 24.65 | | 185.47 ± 17.58 | -31.19 ± 2.24 |
| M17 | DOPE/Egg PC/ DDA  30/60/10 | 0.448 ± 0.093 | 241.90 ± 9.23 | 210.25 ± 27.48 | 175.64 ± 36.72 | | 134.84 ± 22.34 | -28.20 ± 2.29 |
| M18 | DOPE/Egg PC/DDA/ mPEG_2000_-DSPE  37.5/55/2.5/5 | 0.088 ± 0.002 | 112.20 ± 5.91 | 123.50 ± 6.43 | 102.32 ± 4.87 | | 87.42 ± 5.16 | -7.92 ± 0.031 |
| M19 | DOPE/Egg PC/DDA/ mPEG_2000_-DSPE  35/55/5/5 | 0.463 ± 0.09 | 173.30 ± 26.32 | 138.7 ± 24.73 | 117.30 ± 25.23 | | 83.43 ± 13.37 | -10.00 ± 2.93 |
| M20 | DOPE/Egg PC/DDA/ mPEG_2000_-DSPE  32.5/55/7.5/5 | 0.579 ± 0.18 | 169.20 ± 35.42 | 163.30 ± 42.79 | 112.20 ± 38.44 | | 57.82 ± 10.85 | -10.20 ± 1.76 |
| M21 | DOPE/Egg PC/DDA/ mPEG_2000_-DSPE  30/55/10/5 | 0.892 ± 0.20 | 313.5 ± 44.12 | 355.30 ± 47.90 | 66.94 ± 37.23 | | 48.19 ± 42.24 | -10.01 ± 2.10 |
| M22 | DOPE/Egg PC/DDA/ mPEG_2000_-DSPE  37.5/57.5/2.5/2.5 | 0.394 ± 0.16 | 165.70 ± 34.62 | 134.10 ± 19.15 | 120.50 ± 20.00 | | 95.84 ± 43.92 | -15.80 ± 3.80 |
| M23 | DOPE/Egg PC/DDA/ mPEG_2000_-DSPE  35/57.5/5/2.5 | 0.382 ± 0.098 | 152.50 ± 29.14 | 146.10 ± 31.20 | 114.20 ± 30.59 | | 70.39 ± 12.84 | -14.90 ± 2.67 |
| M24 | DOPE/Egg PC/DDA/ mPEG_2000_-DSPE  32.5/57.5/7.5/2.5 | 0.440 ± 0.16 | 150.30 ± 24.34 | 110.70 ± 15.80 | 95.28 ± 15.23 | | 78.19 ± 10.18 | -13.70 ± 4.34 |
| M25 | DOPE/Egg PC/DDA/ mPEG_2000_-DSPE  30/57.5/10/2.5 | 0.516 ± 0.18 | 157.80 ± 21.40 | 101.20 ± 12.76 | 88.68 ± 12.44 | | 75.71 ± 9.11 | -11.40 ± 2.78 |
| M26 | DOPE/Egg PC/DDA/ mPEG_2000_-DSPE  37.5/59/2.5/1 | 0.526 ± 0.24 | 184.5 ± 26.10 | 176.90 ± 32.12 | 92.93 ± 17.10 | | 70.08 ± 13.82 | -17.80 ± 4.42 |
| M27 | DOPE/Egg PC/DDA/ mPEG_2000_-DSPE  35/59/5/1 | 0.570 ± 0.18 | 167 ± 33.48 | 296.50 ± 38.90 | 232.30 ± 35.50 | | 198.19 ± 9.56 | -17.3 ± 3.27 |
| M28 | DOPE/Egg PC/DDA/ mPEG_2000_-DSPE  32.5/59/7.5/1 | 0.369 ± 0.13 | 185.8 ± 27.84 | 238.30 ± 32.69 | 239.80 ± 37.08 | | 46.64 ± 5.00 | -15.10 ± 2.10 |
| M29 | DOPE/Egg PC/DDA/ mPEG_2000_-DSPE  30/59/10/1 | 0.744 ± 0.21 | 207.40 ± 41.23 | 116.70 ± 32.00 | 76.77 ± 22.36 | | 54.85 ± 7.69 | -16.10 ± 2.65 |
| M30 | DOPE/Egg PC/DDA/ mPEG_2000_-DSPE /Chol  27.5/55/2.5/5/10 | 0.118 ± 0.01 | 107.70 ± 6.24 | 120.70 ± 5.64 | 100.30 ± 7.14 | | 76.60 ± 5.96 | -11.30 ± 0.04 |
| M31 | DOPE/Egg PC/DDA/ mPEG_2000_-DSPE /Chol  17.5/55/2.5/5/20 | 0.130 ± 0.009 | 117.40 ± 7.48 | 120.50 ± 6.74 | 108.40 ± 5.98 | | 92.15 ± 6.45 | -11.00 ± 0.042 |
| M32 | DOPE/Egg PC/DDA/ mPEG_2000_-DSPE /Chol  7.5/55/2.5/5/30 | 0.102 ± 0.008 | 123.70 ± 8.21 | 137.90 ± 7.56 | 119.50 ± 6.84 | | 98.64 ± 7.46 | -10.30 ± 0.051 |
| M33 | DOPE/Egg PC/DDA/ mPEG_2000_-DSPE /Chol  0/55/2.5/5/37.5 | 0.141 ± 0.007 | 132.80 ± 6.85 | 147.30 ± 7.41 | | 118.00 ± 8.12 | 100.66 ± 5.63 | -13.10 ± 0.034 |

The difference between M7 and M8 is the external buffer. In M7 formulation the external buffer is phosphate and in the M8 formulation is carbonate.

a, Poly dispersity index

b, Standard deviation

c, Non hydrated

1. **Preparation of RS liposomal formulations containing Pyrvinium Phosphate**

A thin film of DOPE: Egg PC: diselenide: mPEG: Chol with different ratio was prepared and hydrated with PBS containing 1 mg/ml pyrvinium phosphate at 58 °C. After extrusion, sonication and dialysis, the concentration of pyrvinium phosphate was determined using spectrofluorometer (Shimadzu RF5000U, Japan) (ex: 560 nm/em: 680 nm).

**Table S2.** Final RS liposomal formulations after loading of Pyrvinium phosphate

| Name | Formulation | PDI^a^ ± SD^b^ | Z-average ± SD | Intensity ± SD | Volume ± SD | Number ± SD | Zeta Potential ± SD |
| --- | --- | --- | --- | --- | --- | --- | --- |
| M14 | DOPE/Egg PC/ DDA  37.5/60/2.5 | 0.146 ± 0.06 | 183.21 ± 5.77 | 165.37 ± 6.28 | 151.35 ± 5.24 | 123.67 ± 3.83 | -7.84 ± 0.04 |
| M18 | DOPE/Egg PC/DDA/ mPEG_2000_-DSPE  37.5/55/2.5/5 | 0.188 ± 0.02 | 129.90 ± 4.95 | 143.33 ± 4.73 | 128.90 ± 6.37 | 87.72 ± 7.16 | -8.41 ± 0.051 |
| M30 | DOPE/Egg PC/DDA/ mPEG_2000_-DSPE /Chol  27.5/55/2.5/5/10 | 0.096 ± 0.008 | 114.80 ± 5.34 | 127.00 ± 6.61 | 108.20 ± 5.84 | 82.41 ± 4.66 | -10.40 ± 0.038 |
| M31 | DOPE/Egg PC/DDA/ mPEG_2000_-DSPE /Chol  17.5/55/2.5/5/20 | 0.142 ± 0.09 | 133.30 ± 6.86 | 157.20 ± 5.64 | 126.30 ± 5.68 | 80.64 ± 5.75 | -12.80 ± 0.037 |
| M32 | DOPE/Egg PC/DDA/ mPEG_2000_-DSPE /Chol  7.5/55/2.5/5/30 | 0.135 ± 0.03 | 132.70 ± 7.36 | 154.80 ± 6.36 | 128.27 ± 6.54 | 84.18 ± 6.86 | -11.50 ± 0.067 |
| M33 | DOPE/Egg PC/DDA/ mPEG_2000_-DSPE /Chol  0/55/2.5/5/37.5 | 0.134 ± 0.07 | 146.70 ± 5.71 | 157.25 ± 6.91 | 123.09 ± 6.42 | 98.68 ± 5.75 | -8.88 ± 0.049 |

a, Poly dispersity index

b, Standard deviation

c, Non hydrated

**
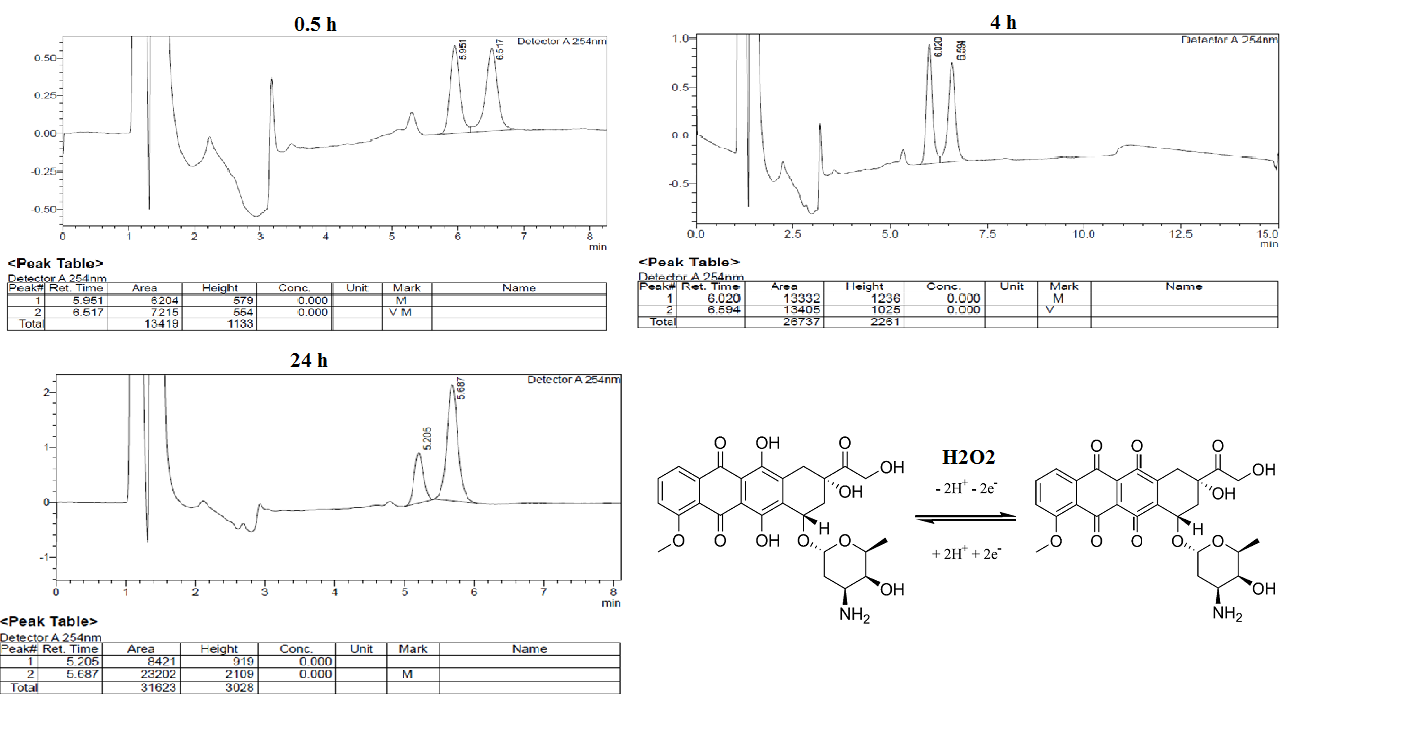
Figure S1.** Oxidation of doxorubicin in presence of H_2_O_2_ after 0.5, 4 and 24h detected by high performance liquid chromatography.
